# Correction: A quantitative modelling approach for DNA repair on a population scale

**DOI:** 10.1371/journal.pcbi.1011602

**Published:** 2023-10-27

**Authors:** The PLOS Computational Biology Staff

Eq 5 is incorrect. The correct equation is:

$$\ln\;\ln\frac{1}{1-f(t)/\theta }=m\;\ln\;t+m\;\ln\;1/\tau .$$

The publisher apologizes for the error.
