# Supplementary material for: A quantitative modelling approach for DNA repair on a population scale
Source: PLoS Comput Biol. 2022 Sep 12;18(9):e1010488. doi: 10.1371/journal.pcbi.1010488 (PMC9499311; doi:10.1371/journal.pcbi.1010488)
Supplement: S1 Appendix — (PDF) [file pcbi.1010488.s001.pdf]

S1 Appendix

Determining TCR Regions. Coordinates for transcribed regions were taken from [1]. This is set to be the TS. The area opposite of the TS is the NTS. All other segments are defined to be intergenic or non-transcribing. Here, we distinguish between Watson (positive) and Crick strand (negative). This allows us to show that there is no strand-specific bias. TCR-regions are defined to be transcripts that repair more or equal to 20% of their initial repair within 20 minutes. Approximately 89% of intergenic regions possess repair rates lower than 20% during the same time span (S2 Fig). Hence, we can have an increased confidence that genic regions with quicker repair are supported by TCR, leading to a more than 1%-point decrease of CPDs per minute. For all other genes, we cannot exclude the possibility that they are only repaired by GGR, as the pathway can function genome-wide. It is important to highlight that the observed CPD decrease is not uniform along the transcript. The end of the TS is seemingly less efficiently repaired. We require that only the first third of the gene after the TSS must possess more than a 20% decrease of damage within 20 minutes to be considered a TCR region.

References

1. Park D, Morris AR, Battenhouse A, Iyer VR. Simultaneous mapping of transcript ends at single-nucleotide resolution and identification of widespread promoter-associated non-coding RNA governed by TATA elements. Nucleic acids research. 2014;42(6):3736–3749.
