# Supplementary material for: A quantitative modelling approach for DNA repair on a population scale
Source: PLoS Comput Biol. 2022 Sep 12;18(9):e1010488. doi: 10.1371/journal.pcbi.1010488 (PMC9499311; doi:10.1371/journal.pcbi.1010488)
Supplement: S2 Appendix — (PDF) [file pcbi.1010488.s002.pdf]

S2 Appendix

Discussing the Effect of Data Transformation and Selection. In order to make our results comparable, we followed the signal analysis described by [1]. However, we used three bins (beginning, centre, and end) instead of six. We also converted the data such that it represents repair instead of damage (Eq 7). This allowed a straightforward application of Eq 3. We additionally required that repair is greater than or equal to zero and monotonously increasing as a function of time (Eq 8). Some studies propose the notion of *dark* or *delayed* CPDs in human cells, which occur after UV treatment [2, 3]. However, to our knowledge there has not been a consensus over how *delayed* CPDs occur and influence repair dynamics. As we assume the biological process as well as the data probing itself to induce a considerable amount of noise, we prefer the interpretation that these data points should be rectified rather than representing damage created after irradiation.

In order to find potential groups that show similar repair dynamics, we compared the distribution of the model parameters against each other. Depending on the chosen segmentation and the type of genomic region, we found two to four clusters which were predominantly determined by the shape parameter m. When investigating the repair dynamics in detail, we found that all groups except one produced a switch-like behaviour (S3B Fig). This is due to the fact that data points cannot be brought into a line (S3C Fig). Whilst this could be a genuine property, we conjecture that this comes from the applied data normalisation. As discussed before, we require that no new lesions can be induced after irradiation. However, almost exclusively all regions with $m > 6.0$ originally possessed larger CPD signals after 20 minutes than directly after irradiation. During the data transformation, this data point was hence set to zero. Due to the form of Eq 3, lesion removal is seemingly acting exclusively between 20 and 60 minutes. Due to the lack of early repair and the data variability, we are convinced that these regions are not repaired by TCR and instead exhibit late acting GGR. They contribute significantly to the two distinguishable mechanisms in Fig 3. However, we have less confidence in the actual parameter values, as we gauge the data correction and consequent step-like behaviour to result from noise. A correlation with other nuclear processes could be therefore significantly biased. We excluded these regions from the

downstream analysis. It should be mentioned that we also tried an amendment to the algorithm to allow a larger flexibility for determining the repair kinetics in these regions. Here, we applied a weighted linear regression and required at least 1%-point difference in repair between two consecutive time steps. However, this solely brings the previously clearly separated groups closely together in parameter space. Consequently, the mutual effect of TCR and GGR becomes difficult to discern (despite being still detectable). There was no major change of the correlation analysis with respect to transcription rate, TU length, and nucleosome density in the *TCR* configuration. However, the relative distance to centromeres and telomeres changed drastically for the *gene* setup. As we have less confidence in the parameter values of regions with large variability, we prefer removing them from the correlation analysis while keeping two distinct repair mechanisms detectable.

In some cases, we could also find a grouping which was driven by the characteristic time τ. Large values only occurred in NTS or non-TCR/non-transcribed regions. We observed that these areas were all comparatively small, i.e. less than 300 base pairs (bp). Therefore, they are very susceptible to noise and processes from neighbouring regions. Instead of requiring a minimal length, we limited the range of τ to keep as many areas with potential useful information as possible. We assumed 200 minutes to be a sufficient time range for CPD repair to occur in yeast. All parameter ranges were set as follows: $m \in [0.5, 6.0]$; $\tau \in [20, 200]$; and $\theta \in [0.5, 1.0]$ for the TS of TCR regions (genes) and $\theta \in [0.4, 1.0]$ for all other areas. The number of remaining regions that fulfilled the set requirements changed considerably depending on the experimental setup. An overview is given in S1 Table. The TS was in almost all cases included in the subsequent analysis, although the end was more often outside the defined parameter ranges than the beginning and centre. Surprisingly, only around half of the NTSs met the requirements (both setups). The numbers are even worse for intergenic/non-TCR regions (both setups). Here, approximately a third of all non-transcribed/non-TCR areas were considered in the downstream computations. We hypothesise that repair at NTS and intergenic regions is dominated by accessinility to the lesion. Late repair times were also reported by [4] and [5]. Considering additionally the few time points, the data fitting of Eq 3 predicts no repair until 20 to 60 minutes, whereas all CPD decrease appears exclusively afterwards. This results in the aforementioned step-like behaviour. As

discussed before, we assume this to be rather unlikely. Analysing the repair dynamics in these regions could provide additional information. We hope that future research is inspired to repeat this analysis with a CPD-seq time course that has a finer temporal resolution.

References

1. Mao P, Smerdon MJ, Roberts SA, Wyrick JJ. Asymmetric repair of UV damage in nucleosomes imposes a DNA strand polarity on somatic mutations in skin cancer. Genome research. 2020;30(1):12–21.

2. Yim S, Lee J, Jo H, Scholten J, Willingham R, Nicoll J, et al. Chrysanthemum morifolium extract and ascorbic acid-2-glucoside (AA2G) blend inhibits UVA-induced delayed cyclobutane pyrimidine dimer (CPD) production in melanocytes. Clinical, cosmetic and investigational dermatology. 2019;12:823.

3. Fajuyigbe D, Douki T, van Dijk A, Sarkany RP, Young AR. Dark cyclobutane pyrimidine dimers are formed in the epidermis of Fitzpatrick skin types I/II and VI in vivo after exposure to solar-simulated radiation. Pigment cell & melanoma research. 2021;34(3):575–584.

4. Li W, Adebali O, Yang Y, Selby CP, Sancar A. Single-nucleotide resolution dynamic repair maps of UV damage in Saccharomyces cerevisiae genome. Proceedings of the National Academy of Sciences. 2018;115(15):E3408–E3415.

5. Mao P, Smerdon MJ, Roberts SA, Wyrick JJ. Chromosomal landscape of UV damage formation and repair at single-nucleotide resolution. Proceedings of the National Academy of Sciences. 2016;113(32):9057–9062.
