# Supplementary material for: A quantitative modelling approach for DNA repair on a population scale
Source: PLoS Comput Biol. 2022 Sep 12;18(9):e1010488. doi: 10.1371/journal.pcbi.1010488 (PMC9499311; doi:10.1371/journal.pcbi.1010488)
Supplement: S3 Appendix — (PDF) [file pcbi.1010488.s003.pdf]

S3 Appendix

Dividing the Data into Genes versus Intergenic Regions. Instead of the commonly used partition into transcripts and intergenic regions, we opted to segment CPD-seq data into TCR (TS and NTS) and non-TCR regions (S1 Fig). In order to increase comparability with existing studies, we repeated the entire analysis for genes (TS and NTS, without sub-areas) and non-transcribed regions (Watson and Crick strand separately). Coordinates for transcripts were taken again from [1]. In the following, this is referred to as *gene* setup. The other aspects of the analysis remain the same.

We observed that the DC is much lower for the *gene* configuration (DC=0.241) (S18A Fig). Nevertheless, this is also true when comparing XR-seq with the repair rate derived from the data (DC=0.231) (S18B Fig). Thus, we judge this to be due to the data segmentation rather than the model assumptions (S4 Appendix) An overview over the correlation values in all setups is given in S2 Table.

When averaging the results over all instances of a region type (e.g. all genes), we can recover once again two distinct dynamics at genes. The early mechanism disappears on the NTS and both strands of intergenic regions, although NTS still shows higher repair rates at early time points than non-transcribed areas. As expected, Watson and Crick strand of intergenic regions follow identical trends. This is in line with our analysis at TCR regions.

Considering the parameter space, the clear pattern of early repair of TCR regions (low values for m) vanishes when considering all genes. The values are scattered much more broadly. As expected, the NTS tends to show low θ values. However, the distribution over m seems to be remarkably similar for all areas, with intergenic regions showing the largest dispersion.

The correlation with biological features differed sometimes considerably, which points out that the type of data segmentation is important. The transcription rate is seemingly weakly correlated with the NTS in the *gene* setup. Though, we should emphasise that both TS and NTS were compared to the same NET-seq value. Hence, we do not consider antisense transcription to be the reason. Instead, this could possibly indicate that accessibility to the lesion is influenced by transcription. Nucleosome density was clearly correlated to Crick and Watson strand in intergenic regions as well

as the TS. There was no link between nucleosome occupancy and repair on the NTS. TU length shows again a very clear interrelationship. Most interestingly, we report that the relative distance to centromeres and telomeres is seemingly important for the TS in the *gene* setup. We are unable to provide an intuition without further biological experiments. However, we could imagine a link between chromosome flexibility and repair, since it enables a larger number of DNA-protein interactions.

References

1. Park D, Morris AR, Battenhouse A, Iyer VR. Simultaneous mapping of transcript ends at single-nucleotide resolution and identification of widespread promoter-associated non-coding RNA governed by TATA elements. Nucleic acids research. 2014;42(6):3736–3749.
