# Supplementary material for: A quantitative modelling approach for DNA repair on a population scale
Source: PLoS Comput Biol. 2022 Sep 12;18(9):e1010488. doi: 10.1371/journal.pcbi.1010488 (PMC9499311; doi:10.1371/journal.pcbi.1010488)
Supplement: S4 Appendix — (PDF) [file pcbi.1010488.s004.pdf]

S4 Appendix

Explaining the Correlation Between XR-seq Data and Repair Rate. As the derivative of Eq 3 represents the repair rate at a given time, we conjectured that it should correlate with XR-seq data. This is due to the fact that the signal shows the distribution of excised nucleotide sequences that were produced during DNA cleavage. It is surmised that they are quickly degraded, i.e. within five minutes [1]. However, this interrelationship is not necessarily linear, which speaks against the usage of Pearson's correlation. The distance correlation (DC) comes as a remedy by relating the distance of data points in a set to each other rather than the data itself (although it should be pointed out that it is not simply the Pearson's correlation of distances). It ranges from zero to one, with zero showing independence, whereas one indicates that the linear subspace between the data sets is equal. It is calculated as follows. Distance matrices A and B contain all pairwise distances, i.e. $\{A\}_{ij} = ||\mathbf{a_i} - \mathbf{a_j}||_2$ and $\{B\}_{ij} = ||\mathbf{b_i} - \mathbf{b_j}||_2$. Here, $\mathbf{a_x}$ and $\mathbf{b_x}$ ($\mathbf{x} \in \{\mathbf{i}, \mathbf{j}\}$) denote data points in sets $\mathcal{A}$ and $\mathcal{B}$, respectively. $||\ldots||_2$ is the Euclidean distance. Each set contains n data points. Subsequently, A and B are double-centred. With the definition of the sample distance covariance

$$\mathrm{dCov}^2(A, B) = \frac{1}{n^2} \sum_j \sum_k A_{jk} B_{jk}, \tag{1}$$

as well as the sample distance variance

$$\mathrm{dVar}(A) = \mathrm{dCov}^2(A, A) = \frac{1}{n^2} \sum_j \sum_k A_{jk}^2, \tag{2}$$

,

we can introduce the DC:

$$DC(\mathcal{A}, \mathcal{B}) = \frac{\mathrm{dCov}^2(A, B)}{\sqrt{\mathrm{dVar}(A)\mathrm{dVar}(B)}}. \tag{3}$$

The DCs for all setups are given in S2 Table. In order to compare the values of Eq 6 to the CPD data, we transformed first the signal with respect to Eq 7. We calculated relative repair at three time points (i.e. 20, 60 and 120 minutes) by

$$r(t'_i) = \frac{R(t_i) - R(t_{i-1})}{(t_i - t_{i-1})/20}, \tag{4}$$

where $R(t)$ denotes repair determined by Eq 7. $t_i \in \mathbf{t} = (20, 60, 120)$ and $t'_i \in \mathbf{t'} = (5, 20, 60)$. The values must be re-scaled to the same time step to make them comparable. However, the CPD decrease within the first 20 minutes is relatively small for most areas. Calculating $r(t)$ per minute results in an almost flat line. All values represent therefore repair within 20 minutes. This makes the time points comparable whilst avoiding having too small values for $r(5)$.

Despite the fact that we determined the DC for all time points—i.e. 5, 20, and 60 minutes after repair—it is intuitive to see that $r(t)$ is more heterogeneous when taking all time points together. We therefore consider only the DC of the entire data set.

References

1. Li W, Adebali O, Yang Y, Selby CP, Sancar A. Single-nucleotide resolution dynamic repair maps of UV damage in Saccharomyces cerevisiae genome. Proceedings of the National Academy of Sciences. 2018;115(15):E3408–E3415.
