# Supplementary material for: A quantitative modelling approach for DNA repair on a population scale
Source: PLoS Comput Biol. 2022 Sep 12;18(9):e1010488. doi: 10.1371/journal.pcbi.1010488 (PMC9499311; doi:10.1371/journal.pcbi.1010488)
Supplement: S5 Appendix — (PDF) [file pcbi.1010488.s005.pdf]

S5 Appendix

Discussing the kNN Approach. The parameters of Eq 3 can be used to set repair into the context of other biological and nuclear properties in the cell. As databases provide a large variety of data signals, it is reasonable to opt for a data driven approach. However, finding such a correlation is not straightforward. Many of the NGS histogram distributions peak sharply around a low value whilst also including far distant outliers. Moreover, it can be assumed that sequencing signals include a large amount of noise. Precise predictions for data values around the histogram peak are difficult. This excludes continuous regression approaches or widely used correlation indices, such as Pearson's correlation, DC, or mutual information. We circumvented these issues by transforming the mapping into a binary classification problem to analyse general trends. This reduces the impact of noise. Due to using equally many values for both classes, we removed any distribution specific bias.

Another requirement was comparability between results. It is a known fact that the performance of machine learning models can vary strongly depending on the number of parameters or used architecture [1]. Through using the nonparametric kNN approach, we could provide equivalent treatment for all setups. We also mitigated the impact of k by applying different values in a reasonable range, i.e. $k \in \{5, 10, 20, 50, 100\}$. Thus, we did not rely on any particular parameter setting or defined spline ranges. We are aware that parametric models can be efficiently implemented for a variety of tasks, as it has been recently shown with Alpha Fold 2 [2]. Moreover, architectural biases of parametric models could be possibly reduced by systematic parameter searches. Nevertheless, it should be emphasised that this study did not intend to find the best performing machine learning model. Rather, we aimed to show non-random correlations and therefore indicate potential repair influences. Some researchers even conjecture that nonparametric models could be generally better performing [3]. We conclude that the kNN approach is a sensible choice.

In the following, we want to provide some further intuition using the example of TU length. S6 Fig shows the learnt function and the prediction error distribution for the correct and random model, respectively. The correct mapping finds a distribution pattern with big genes predominantly distributed to the centre right, whereas small

genes are found in its periphery (S6A Fig). As expected, this pattern is destroyed in the random mapping (S6C Fig). The error distribution for the true kNN is equally large for values that are over and underestimated (right histogram in S6B Fig). This speaks for an unbiased mapping. We also want to point attention towards the spatial distribution of the prediction error. Red circles mark data values which were incorrectly classified as large genes, whereas the blue points are parameters that were wrongly associated with a small size. The distribution of the red and blue disks follows our expectations from the learnt map. The wrong classification could indicate noise in the data set or unknown information that cannot be represented. Compared with S6D Fig, it is clear that these trends vanish in the random model. However, the prediction error is surprisingly low (0.4). A repetition over 100 iterations is therefore indispensable to find significant links and to remove data specific biases.

References

1. Dyson F. A meeting with Enrico Fermi. Nature. 2004;427(6972):297–297.
2. Jumper J, Evans R, Pritzel A, Green T, Figurnov M, Ronneberger O, et al. Highly accurate protein structure prediction with AlphaFold. Nature. 2021;596(7873):583–589.
3. Perretti CT, Munch SB, Sugihara G. Model-free forecasting outperforms the correct mechanistic model for simulated and experimental data. Proceedings of the National Academy of Sciences. 2013;110(13):5253–5257.
