# Supplementary material for: A quantitative modelling approach for DNA repair on a population scale
Source: PLoS Comput Biol. 2022 Sep 12;18(9):e1010488. doi: 10.1371/journal.pcbi.1010488 (PMC9499311; doi:10.1371/journal.pcbi.1010488)
Supplement: S6 Appendix — (PDF) [file pcbi.1010488.s006.pdf]

S6 Appendix

Analysing Repair Kinetics in Context of Abf1 and H2A.Z Distribution. Different chromatin features including transcription factor binding sites or histone variants and modifications can affect CPD repair. An essential role in GGR recognition is allocated to the Rad7-Rad16-Abf1 protein complex. Yeast strains with respective gene deletions are incapable to repair lesions in non-transcribed regions and are inviable under genotoxic stress [1, 2, 3]. Abf1 binding was also proposed to inhibit CPD formation [4] and to influence GGR kinetics [2, 3]. Moreover, TCR and GGR are both reportedly influenced by multiple histone modifications and variants [5]. Binding sites for the hypothesised GGR-complex are flanked by H2A.Z histone variant-containing nucleosomes [6]. However, a direct relationship between lesion removal and Abf1 occupancy or H2A.Z distribution has not been investigated. Building up on previous work, we presumed particularly strong correlation in intergenic regions. Abf1 and H2A.Z distribution was probed after UV treatment by [6]. In the following, we include results from the *TCR* configuration (as presented in the main text) and the *gene* setup (see S3 Appendix).

The experiments with Abf1 yielded a mixed bag of results (S10 Fig). When considering the *TCR* setup, we found a non-random correlation with the repair dynamics in non-TCR areas regions for all k (S5 Appendix). A strong correlation is in line with the idea of Abf1 being part of the GGR complex, whose effect is presumed to be strong in non-TCR regions [6]. The end of TCR areas seemed to exhibit a slight correlation with Abf1 as well. However, the *gene* configuration found a link to almost all regions with the exception of the Watson strand in intergenic areas. This is surprising, as we would expect both strands to behave similarly. Whilst this could hint to a strand-specific bias, it is likely that the influence of Abf1 at intergenic regions in the *gene* setup is weaker than in the *TCR* configuration. A correct definition of genomic areas is hence clearly important to put the results into the right context. Abf1's role is associated with GGR [2, 3, 6] as well as transcription regulation and replication [7, 8]. It is intuitive that due to its multifunctional involvement, it is indirectly affecting a broad variety of regions.

The outcomes for H2A.Z were similarly ambiguous (S11 Fig). We found a

non-random correlation to all regions in the *TCR* configuration. On the other hand, we were unable to find a significant correlation in intergenic areas for the *gene* setup. Nonetheless, there was a definite interrelationship between the histone marker distribution and the repair dynamics at the TS and NTS. This indicates a non-negligible role for H2A.Z modification during lesion removal at active genes. This is unsurprising giving its regulatory role in gene expression [9]. However, a correlation with GGR might be less strong.

To put the results into context, it is important to mention that the histogram distribution of Abf1 and H2A.Z data was different in intergenic regions compared to transcribed areas. This was especially visible in the *gene* setup. It has been previously reported that Abf1 binding sites tend to colocalise with CID boundaries, which are usually found in intergenic regions. In the same paper, it was also proposed that they are flanked by H2A.Z-containing barrier nucleosomes [6]. Assuming that Abf1 is necessary for GGR [1, 3]—and therefore plays a specifically crucial role in intergenic regions—it is not surprising that the histogram exhibits different distributions for genes and non-transcribed areas. Since the distributions are still similar between strands, we do not consider this as having a strong influence on the final conclusions.

References

1. Boiteux S, Jinks-Robertson S. DNA repair mechanisms and the bypass of DNA damage in Saccharomyces cerevisiae. Genetics. 2013;193(4):1025–1064.

2. Yu S, Owen-Hughes T, Friedberg EC, Waters R, Reed SH. The yeast Rad7/Rad16/Abf1 complex generates superhelical torsion in DNA that is required for nucleotide excision repair. DNA repair. 2004;3(3):277–287.

3. Yu S, Evans K, Van Eijk P, Bennett M, Webster RM, Leadbitter M, et al. Global genome nucleotide excision repair is organized into domains that promote efficient DNA repair in chromatin. Genome research. 2016;26(10):1376–1387.

4. Mao P, Smerdon MJ, Roberts SA, Wyrick JJ. Chromosomal landscape of UV damage formation and repair at single-nucleotide resolution. Proceedings of the National Academy of Sciences. 2016;113(32):9057–9062.

5. Adam S, Dabin J, Polo SE. Chromatin plasticity in response to DNA damage: The shape of things to come. DNA repair. 2015;32:120–126.

6. van Eijk P, Nandi SP, Yu S, Bennett M, Leadbitter M, Teng Y, et al. Nucleosome remodeling at origins of global genome–nucleotide excision repair occurs at the boundaries of higher-order chromatin structure. Genome research. 2019;29(1):74–84.

7. Yarragudi A, Miyake T, Li R, Morse RH. Comparison of ABF1 and RAP1 in chromatin opening and transactivator potentiation in the budding yeast Saccharomyces cerevisiae. Molecular and cellular biology. 2004;24(20):9152–9164.

8. Kohzaki H, Murakami Y. A transcription factor Abf1 facilitates ORC binding onto the Saccharomyces cerevisiae replication origin via histone acetylase Gcn5. bioRxiv. 2019; p. 583310.

9. Giaimo BD, Ferrante F, Herchenröther A, Hake SB, Borggrefe T. The histone variant H2A. Z in gene regulation. Epigenetics & chromatin. 2019;12(1):1–22.
