# Supplementary material for: A quantitative modelling approach for DNA repair on a population scale
Source: PLoS Comput Biol. 2022 Sep 12;18(9):e1010488. doi: 10.1371/journal.pcbi.1010488 (PMC9499311; doi:10.1371/journal.pcbi.1010488)
Supplement: S7 Appendix — (PDF) [file pcbi.1010488.s007.pdf]

S7 Appendix

Comparing the KJMA Model With Other Approximations. The KJMA model is used as a tool to find the parameters. It remains to address that the representation is reasonable. We compared the accuracy of Eq 3 with a linear model as well as a logistic regression and the Hill equation, both of which produce an S-shaped function. Performance was measured using the mean-squared error

$$\text{MSE} = \frac{1}{n}\sum_{i=1}^{n}\left(Y_i - \hat{Y}_i\right)^2. \tag{1}$$

An example is given in S13B Fig. We can confirm that the KJMA model achieves a lower error than the linear or the logistic regression model. However, the Hill equation seems to perform slightly yet significantly better (S13C Fig). Therefore, it seems likely that the desired function should follow an S-shape trajectory that is non-symmetric at the inflection point (in contrast to the logistic regression).

We want to provide some mathematical explanation why the Hill function performs similar yet slightly better. Assuming a ligand concentration [L], the fraction of bound receptor proteins can be described by

$$f(t) = \frac{1}{1 + \left(\frac{K_A}{[\text{L}]}\right)^{m'}},$$

where K_A is the ligand concentration that is resulting in $f(t) = 0.5$, and m' represents the number of binding sites on the target protein. When assuming $\left(\frac{K_A}{[\text{L}]}\right)^{m'}$ to be small—i.e. there is a sufficient surplus of the ligand L—we can approximate the Hill equation by $f(t) \approx 1 - \left(\frac{[\text{L}]}{K_A}\right)^{m'}$. This is nothing else but the first order Taylor expansion of $1 - \exp\left[\left(-\frac{[\text{L}]}{K_A}\right)^{m'}\right]$, which has the same form as the KJMA model. The slightly different behaviour is due to the shape of $1/x$ (which is the determining term in the Hill equation) and $1 - e^{-x}$.

It should be stressed that the Hill equation does not have a particular meaning to describe a temporal process such as DNA repair, since it was used to explain ligand occupancy with respect to the available quantity. There is no notion of time. We also noticed that when taking the average over regions that were supposed to have no TCR, the Hill equation still showed a double-repair behaviour (S13D Fig). Together with the

fact that both perform similarly, we think that the KJMA model provides a good representation.
