# Supplementary material for: A quantitative modelling approach for DNA repair on a population scale
Source: PLoS Comput Biol. 2022 Sep 12;18(9):e1010488. doi: 10.1371/journal.pcbi.1010488 (PMC9499311; doi:10.1371/journal.pcbi.1010488)
Supplement: S8 Appendix — (PDF) [file pcbi.1010488.s008.pdf]

S8 Appendix

Discussing the Model in Context of the Physical KJMA Model.

The KJMA model itself has been successfully applied in a biological context to analyse the dynamics of DNA replication in eukaryotes [1]. Nevertheless, the study did not include predictions with respect to independently probed data. More importantly, it is specific for DNA replication and cannot be applied to analyse CPD repair. In order to allow a physical interpretation and to re-group repair regions to patterns, we presumed independent repair kinetics between and within cells. Independence between cells has been discussed already above. The supposition of independent repair dynamics within a cell is based on two observations. Firstly, we assume that the spatial effect of lesion removal kinetics decreases as a function of distance. Hence, the farther away the CPD positions, the smaller the impact on each other. This is justified by the relatively small area of lesion removal ($\approx$ 30 nt [2]) and the notion of chromosome interaction domains (CIDs) [3]. Secondly, it has been reported for *Caenorhabditis elegans* that a UVC treatment of 100J/m^2 induces 0.4 to 0.5 CPDs per 10kb [4]. A similar UV dose (125 J/m^2) was used by [5]. Taking this as a reference, we presume that a comparable dose of UVC induces a corresponding number of CPDs in budding yeast. It is thus unreasonable to expect more than one lesion per CID per cell, as they are commonly less than 10kb in *Saccharomyces cerevisiae* [3]. It is true though that this could be species-dependent. Due to the lack of other studies, we take it as given that lesion removal does not affect each other within a single cell.

The independence assumptions permitted the application of the KJMA model. As we surmise that the sequencing data contains a hidden axis, we represent repair on a grid. Thus, it would be expected that the found shape parameter indicates a two-dimensional space, i.e. $m - 1 \approx 2$. However, as reported above, m exhibits a large range. We do not presume that such a deviation is only caused by noise. Instead, a similar behaviour can be observed when allowing the growth speed G to be larger during earlier time points rather than later in the process, and vice versa. Implicitly, this incorporates the possibility that $G(t)$ is non-constant in time. m can be interpreted to speak for the time-dependence of the process instead of a particular dimension [6]. Low values represent quicker repair in the beginning rather than in the end. A large m

indicates that G increases later on.

The nucleation rate n though is presumed to be constant. As explained above, this has as a consequence that the framework models repair with only one mechanism per region. There is a scientific consensus that intergenic regions and the NTS can be only repaired by GGR. For the TS of genes it is nevertheless surmised that TCR and GGR can act collectively. On a population scale, this would likely result in the repair rate to contain two peaks over time. TCR would be observable in the beginning and subsequently abate. GGR is supposedly acting later during the process. The collective effect of TCR and GGR in a genic area can be recovered by taking the average over an entire group, e.g. the beginning of TCR regions. Despite assuming similar kinetics, we presume that the noise in the process should lead to a representation by either TCR or GGR in a ratio comparable to their respective repair contribution. It should be highlighted that Eq 3 can be easily adapted to represent heterogeneous repair times by defining $n(t)$ (and therefore $\beta(t)$ since $n(t)$ is incorporated) as a function of time. However, any parameter estimation of such a function would be merely based on guesses due to the sparse temporal data resolution. We followed the principle of Occam's razor and opted for a simpler model. The production of CPD data with smaller time steps could permit such an estimation.

Finally, we also want address the analogy of the KJMA model to the stochastic point process. We linked the expansion of the pattern— and therefore $G(t)$—to the diffusion in our model. This can be explained by considering Eq 4, as it includes the Avrami exponent that we before linked to the time dependence of the process. From the perspective of the stochastic point process, the time dependence is incorporated by the diffusion, whose mean squared displacement is proportional to $D_{\hat{m}} t^{\hat{m}}$. The nucleation rate n was compared to the expected waiting time $\hat{\beta}$, both of which are time-independent. Consequently, the growth of repair patterns in the abstract repair space becomes an important property, since if we would only consider a constant nucleation rate, the resulting repair dynamics should follow the trajectory of a homogeneous Poisson point process with constant λ (S13 Fig).

We want to highlight that this alternative understanding cannot be taken literally and should be therefore used with some scepticism. However, we strongly believe that this interpretation could potentially unlock additional information, as it allows the

incorporation of well studied results from the physical model.

References

1. Jun S, Bechhoefer J. Nucleation and growth in one dimension. II. Application to DNA replication kinetics. Physical Review E. 2005;71(1):011909.

2. Boiteux S, Jinks-Robertson S. DNA repair mechanisms and the bypass of DNA damage in Saccharomyces cerevisiae. Genetics. 2013;193(4):1025–1064.

3. Hsieh THS, Weiner A, Lajoie B, Dekker J, Friedman N, Rando OJ. Mapping nucleosome resolution chromosome folding in yeast by micro-C. Cell. 2015;162(1):108–119.

4. Meyer JN, Boyd WA, Azzam GA, Haugen AC, Freedman JH, Van Houten B. Decline of nucleotide excision repair capacity in aging Caenorhabditis elegans. Genome biology. 2007;8(5):1–17.

5. Mao P, Smerdon MJ, Roberts SA, Wyrick JJ. Chromosomal landscape of UV damage formation and repair at single-nucleotide resolution. Proceedings of the National Academy of Sciences. 2016;113(32):9057–9062.

6. Christian JW. The theory of transformations in metals and alloys. Newnes; 2002.
