# Supplementary material for: A quantitative modelling approach for DNA repair on a population scale
Source: PLoS Comput Biol. 2022 Sep 12;18(9):e1010488. doi: 10.1371/journal.pcbi.1010488 (PMC9499311; doi:10.1371/journal.pcbi.1010488)
Supplement: S9 Appendix — (PDF) [file pcbi.1010488.s009.pdf]

S9 Appendix

Investigating a Link Between Transcription Rate and TU length. It is usually assumed that the size of genes does not influence the frequency with which they are transcribed. Thus, both parameters are expected to be independent. During our analysis, we noticed that the NET-seq signal amplitude decreases as function of distance from the TSS (S15 Fig). This could possibly induce a size-specific bias if the decline occurs repeatedly within a specific distance, e.g. 500 bp from the TSS. In this case, it would affect small genes more strongly than large genes. In order to gauge the bias' impact, we compared the NET-seq signal with Pol2 ChIP-seq data [1], which we assume to represent transcription rate to a reasonable degree. We can verify that Pol2 ChIP-seq does not exhibit the same declining trend after the TSS. Indeed, NET-seq shows a larger correlation with respect to TU length (DC=0.321) than Pol2 occupancy (DC=0.224, S16 Fig). However, we can establish a rather strong interrelationship between NET-seq and Pol2 ChIP-seq data (DC=0.75, S17 Fig). When we scrutinised the link to repair, we divided all genes into two groups with high or low transcription rate. We can verify that the majority is still within the same group, independent of the use of NET-seq or Pol2 ChIP-seq data. Therefore, the conclusions about the relationship between transcription and repair remain nevertheless sensible. We opted to use NET-seq data to permit a direct comparison with the results from [2].

References

1. Georges A, Gopaul D, Denby Wilkes C, Giordanengo Aiach N, Novikova E, Barrault MB, et al. Functional interplay between Mediator and RNA polymerase II in Rad2/XPG loading to the chromatin. Nucleic acids research. 2019;47(17):8988–9004.

2. Li W, Adebali O, Yang Y, Selby CP, Sancar A. Single-nucleotide resolution dynamic repair maps of UV damage in Saccharomyces cerevisiae genome. Proceedings of the National Academy of Sciences. 2018;115(15):E3408–E3415.
