# Supplementary material for: A quantitative modelling approach for DNA repair on a population scale
Source: PLoS Comput Biol. 2022 Sep 12;18(9):e1010488. doi: 10.1371/journal.pcbi.1010488 (PMC9499311; doi:10.1371/journal.pcbi.1010488)
Supplement: S1 Table — IGR abbreviates intergenic regions. (PDF) [file pcbi.1010488.s010.pdf]

S1 Table

The number of models per region, before and after applying the requirements for parameter ranges. IGR abbreviates intergenic regions.

Experimental setup	Region name	#Total	#Filtered
Gene	TS	4973	4356
	NTS	4973	2294
	IGR +	4067	1591
	IGR -	4067	1583
TCR	TS start	1878	1865
	TS centre	1878	1703
	TS end	1878	1367
	NTS start	1878	840
	NTS centre	1878	1080
	NTS end	1878	1117
	IGR	1763	650
