# Supplementary material for: A quantitative modelling approach for DNA repair on a population scale
Source: PLoS Comput Biol. 2022 Sep 12;18(9):e1010488. doi: 10.1371/journal.pcbi.1010488 (PMC9499311; doi:10.1371/journal.pcbi.1010488)
Supplement: S2 Table — (PDF) [file pcbi.1010488.s011.pdf]

S2 Table

The DC between XR-seq and repair predictions / data for different experimental configurations.

Experimental setup	5 min	20 min	60 min	Total
TCR setup: **model**	0.405	0.525	0.258	0.441
TCR setup: **data**	0.433	0.644	0.452	0.209
Gene setup: **model**	0.226	0.396	0.216	0.241
Gene setup: **data**	0.242	0.621	0.342	0.231
