# Supplementary material for: A quantitative modelling approach for DNA repair on a population scale
Source: PLoS Comput Biol. 2022 Sep 12;18(9):e1010488. doi: 10.1371/journal.pcbi.1010488 (PMC9499311; doi:10.1371/journal.pcbi.1010488)
Supplement: S3 Table — The table gives the number of kNN models that could find a correlation between model parameters and genomic context. k ∈ {5, 10, 20, 50, 100}. We defined a link to be significant if at least three out of five k find a non-random interrelationship.—means that data was not used in the given configuration. NET denotes NET-seq data, ND is nucleosome density, and meres give the relative distance to centromeres or telomeres. Suffixes S, C, and E denote start, centre and end of an area. NTCR are non-TCR areas. IGR are intergenic/non-transcribed regions. (PDF) [file pcbi.1010488.s012.pdf]

S3 Table

Number of non-random interrelationships between model parameters and sequencing data over k. The table gives the number of kNN models that could find a correlation between model parameters and genomic context. $k \in \{5, 10, 20, 50, 100\}$. We defined a link to be significant if at least three out of five k find a non-random interrelationship. - means that data was not used in the given configuration. NET denotes NET-seq data, ND is nucleosome density, and meres give the relative distance to centromeres or telomeres. Suffixes S, C, and E denote start, centre and end of an area. NTCR are non-TCR areas. IGR are intergenic/non-transcribed regions.

	***TCR* setup**							***Gene* setup**			
	TS S	TS C	TS E	NTS S	NTS C	NTS E	NTCR	TS	NTS	IGR+	IGR-
NET	5	5	5	0	1	1	-	5	3	-	-
Size	5	5	5	5	5	5	-	5	5	-	-
ND	3	2	0	0	0	0	5	5	0	5	5
Abf1	0	2	3	0	0	0	5	4	5	2	4
H2A.Z	5	5	5	5	5	4	3	5	5	2	0
Meres	0	0	0	0	0	0	0	5	0	0	0
