# Supplementary figures and images for: A quantitative modelling approach for DNA repair on a population scale
Source: PLoS Comput Biol. 2022 Sep 12;18(9):e1010488. doi: 10.1371/journal.pcbi.1010488 (PMC9499311; doi:10.1371/journal.pcbi.1010488)

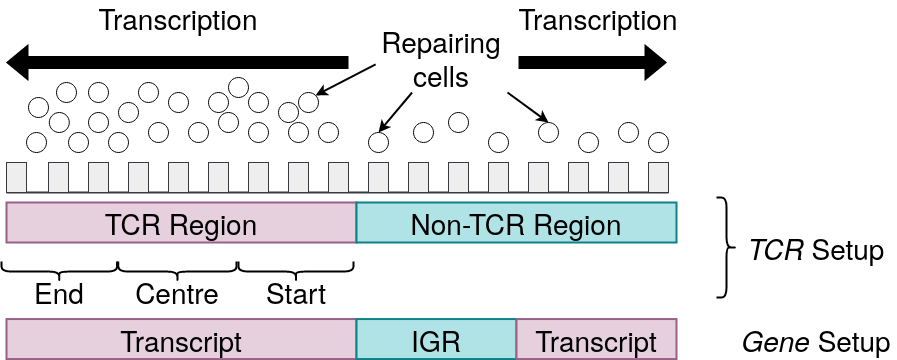

Supplement: S1 Fig — The circles represent the number of cells with ongoing repair in the region. The arrows indicate the region and direction of transcription. The results in the paper follow the TCR setup. Here, only the first gene is considered as TCR area which shows more efficient repair than intergenic regions within the first 20 minutes after UV irradiation. All other parts are labelled as non-TCR region. Therefore, it spans from the end of the first gene to the end of the second. The gene configuration (S3 Appendix) partitions the genome into the traditional notion of transcribed and intergenic regions. The TU positions were determined by [29]. (TIF) [file pcbi.1010488.s013.tif]

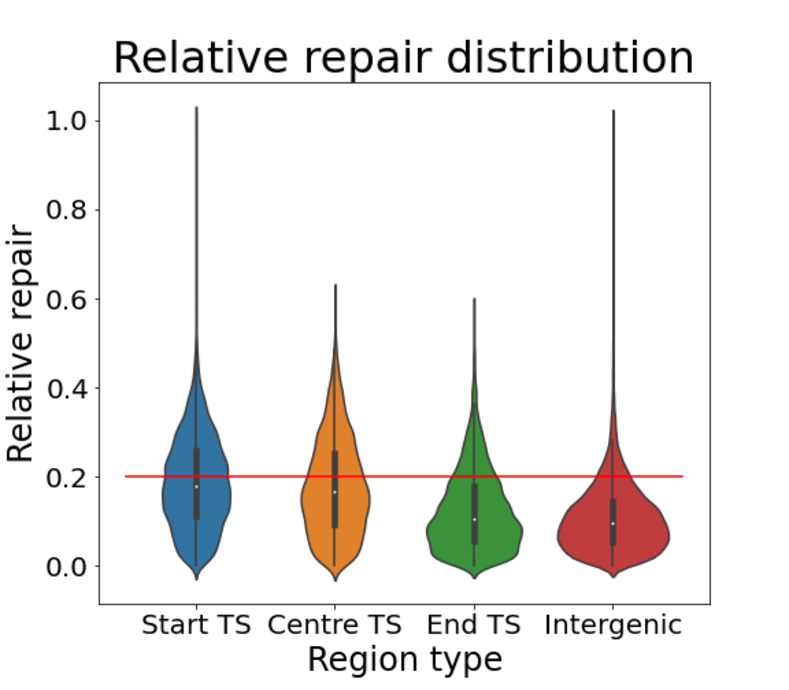

Supplement: S2 Fig — Relative repair in non-transcribed regions is chiefly lower than 20% within the first 20 minutes (88.95%). Genic areas with stronger repair dynamics are thus likely supported by TCR. For all other transcripts, we cannot exclude the possibility that they are exclusively repaired by GGR. (TIF) [file pcbi.1010488.s014.tif]

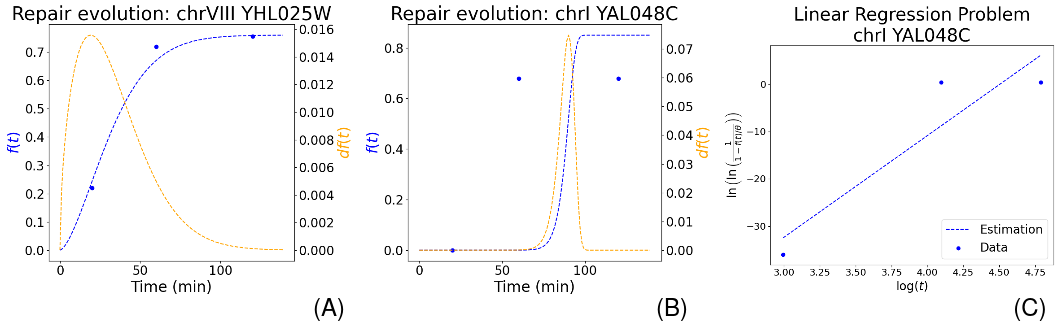

Supplement: S3 Fig — Data points are given by solid dots. The blue dashed line represents the repair fraction predicted by the model (left axis). The orange dashed line shows the derivative (right axis). (A) The SNF6 gene can be well approximated. (B) However, GEM1 exhibits no repair within the first 20 minutes, which results in a switch-like behaviour. (C) This is better understood when showing the data points after transformation according to Eq 5. A linear regression is difficult since they do not align. (TIF) [file pcbi.1010488.s015.tif]

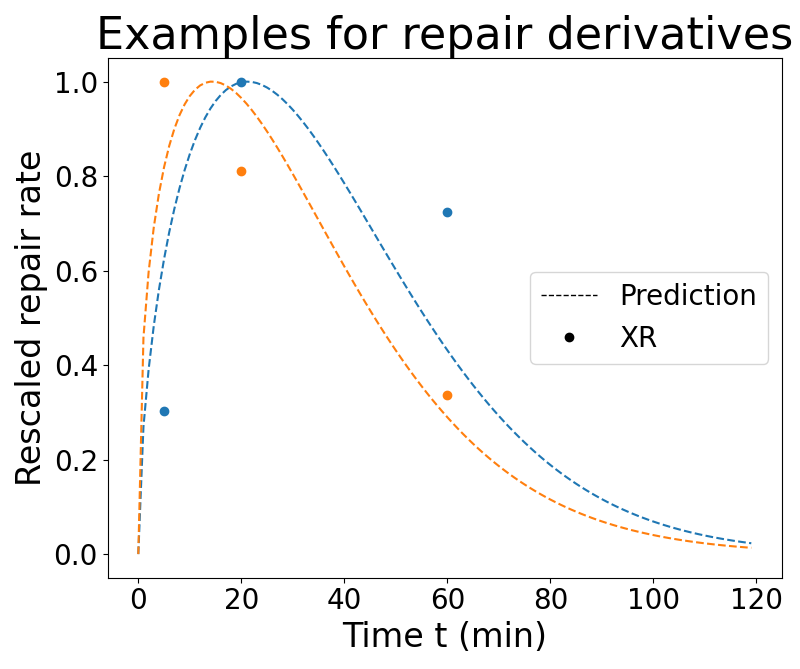

Supplement: S4 Fig — XR-seq data (points) and the predicted repair rate (dashed lines) are exemplified for genes BDH1 (orange) and BDH2 (blue). When re-scaling XR data and repair rate prediction between 0 and 1, both follow clearly similar trends. BDH2 has its largest XR-seq value at 20 minutes post-irradiation, whereas BDH1 shows biggest repair rates after 5 minutes. This is indeed captured by the model. (TIF) [file pcbi.1010488.s016.tif]

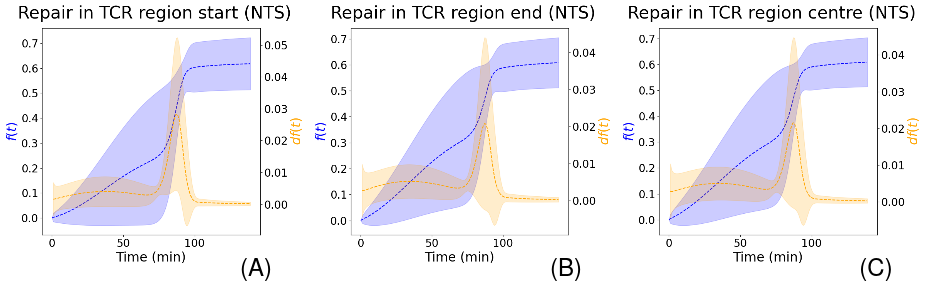

Supplement: S5 Fig — The average repair evolution for the NTS (blue dashed line) shows a much lower repair fraction (≈0.6) than the other areas. Moreover, the repair rate (orange dashed line) indicates repair at early time points. This could be caused by possible overlapping transcripts or by antisense-transcription-coupled repair. Shaded areas show the standard deviation. The repair trajectory is the same for (A) the beginning, (B) the centre, and (C) the end of the NTS. (TIF) [file pcbi.1010488.s017.tif]

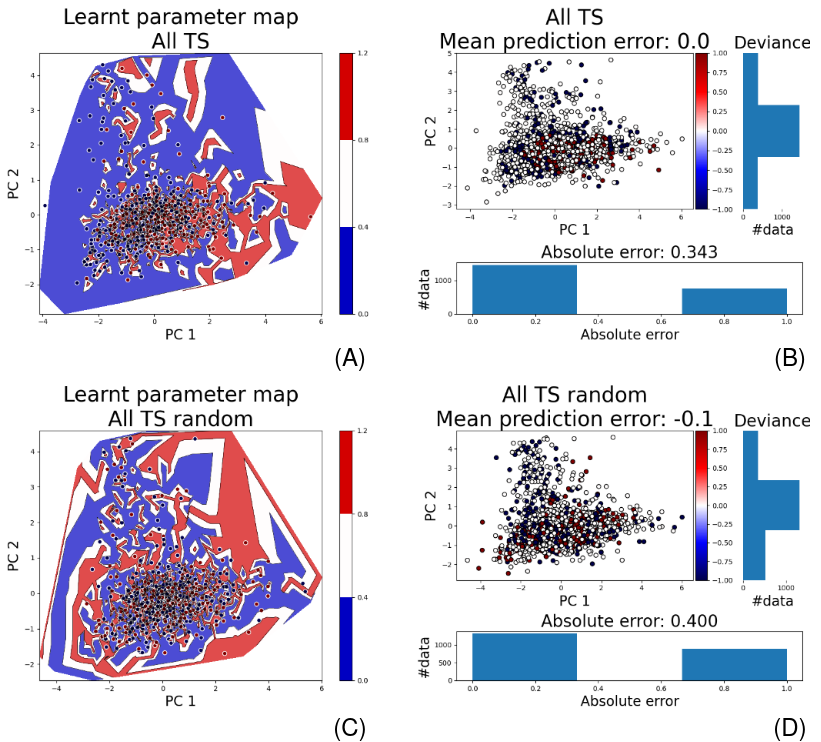

Supplement: S6 Fig — (A) The learnt parameter distribution and the associated class for the true model after applying a principle component transformation. The x and y-axis give the first and second principle component, respectively. Red represent large genes, whereas blue shows low values. (B) The error distribution for the predictions follows the expected outline given by the learnt function in (A). The blue and red circles give values that were classified as short but were actually large and vice versa, respectively. White points are correctly classified. The right bar shows the error distribution along the colour axis, i.e. over estimated, correctly classified, and underestimated values from top to bottom. The lower histogram shows the distribution of overall correctly and incorrectly classified values. (C, D) The learnt parameter map and the error distribution of the random model. (TIF) [file pcbi.1010488.s018.tif]

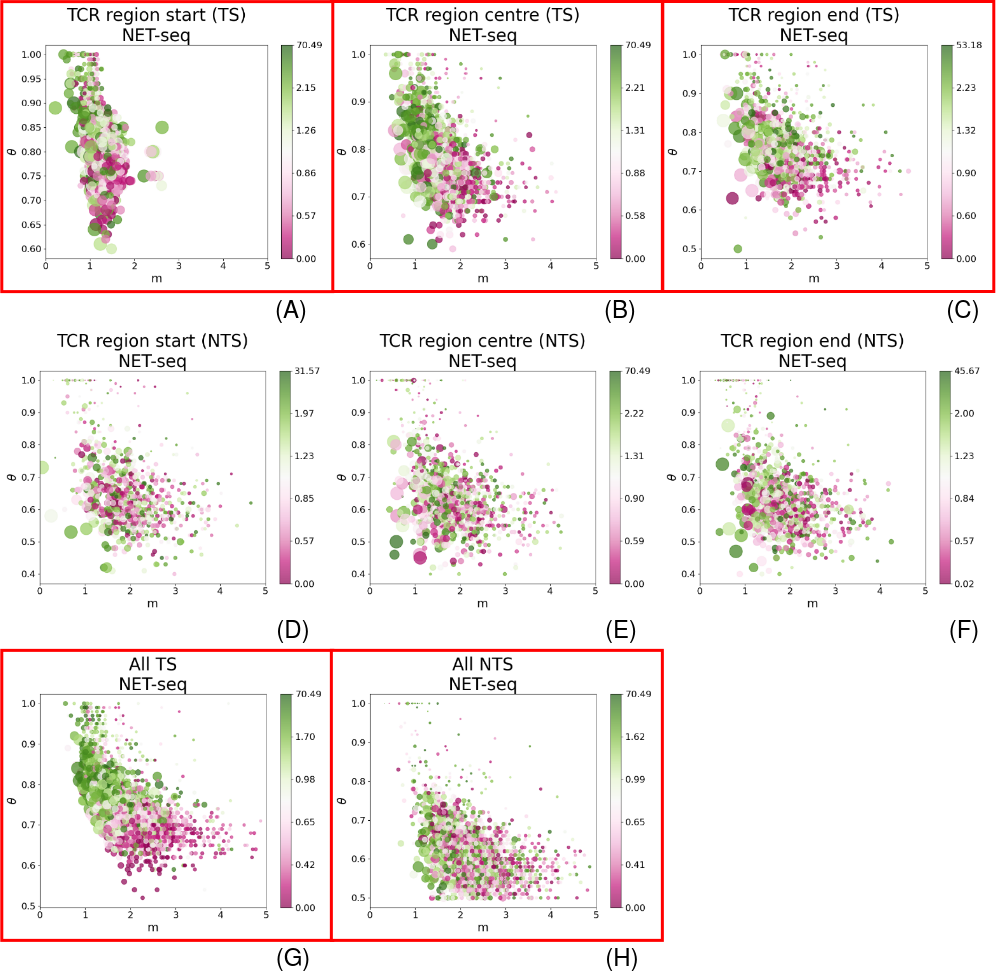

Supplement: S7 Fig — Our results for the transcription rate support the hypothesis that it influences repair on the TS. The x and y-axis give the values of m and θ, respectively. The size of the circles show 1/τ: the larger the circle, the shorter the characteristic time. Significant interrelationships are marked with a red frame. (TIF) [file pcbi.1010488.s019.tif]

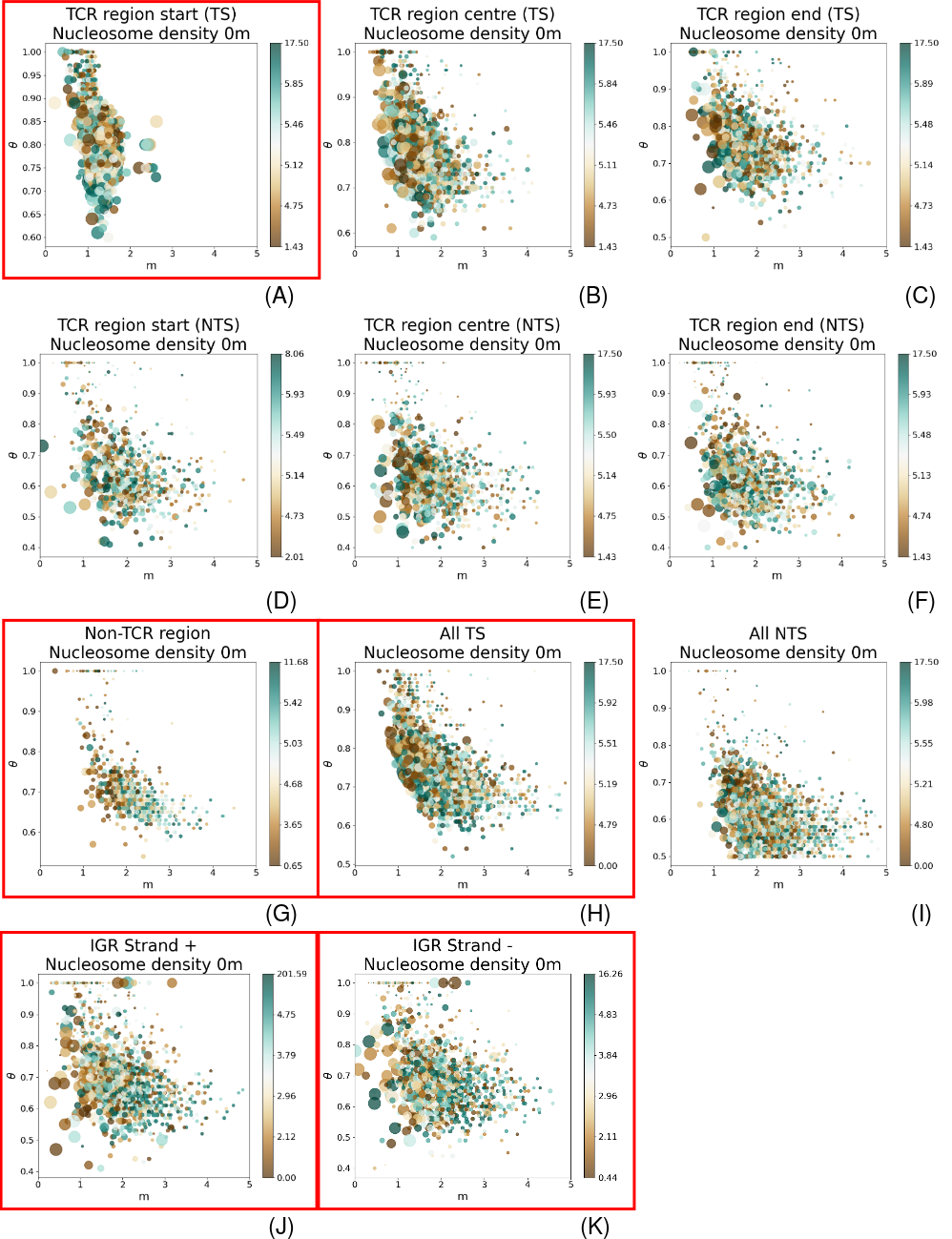

Supplement: S8 Fig — Nucleosome density is seemingly influencing repair in non-transcribed/non-TCR regions as well as the beginning of the TCR TS and the TS in the gene setup. The x and y-axis give the values of m and θ, respectively. The size of the circles show 1/τ: the larger the circle, the shorter the characteristic time. Significant interrelationships are marked with a red frame. (TIF) [file pcbi.1010488.s020.tif]

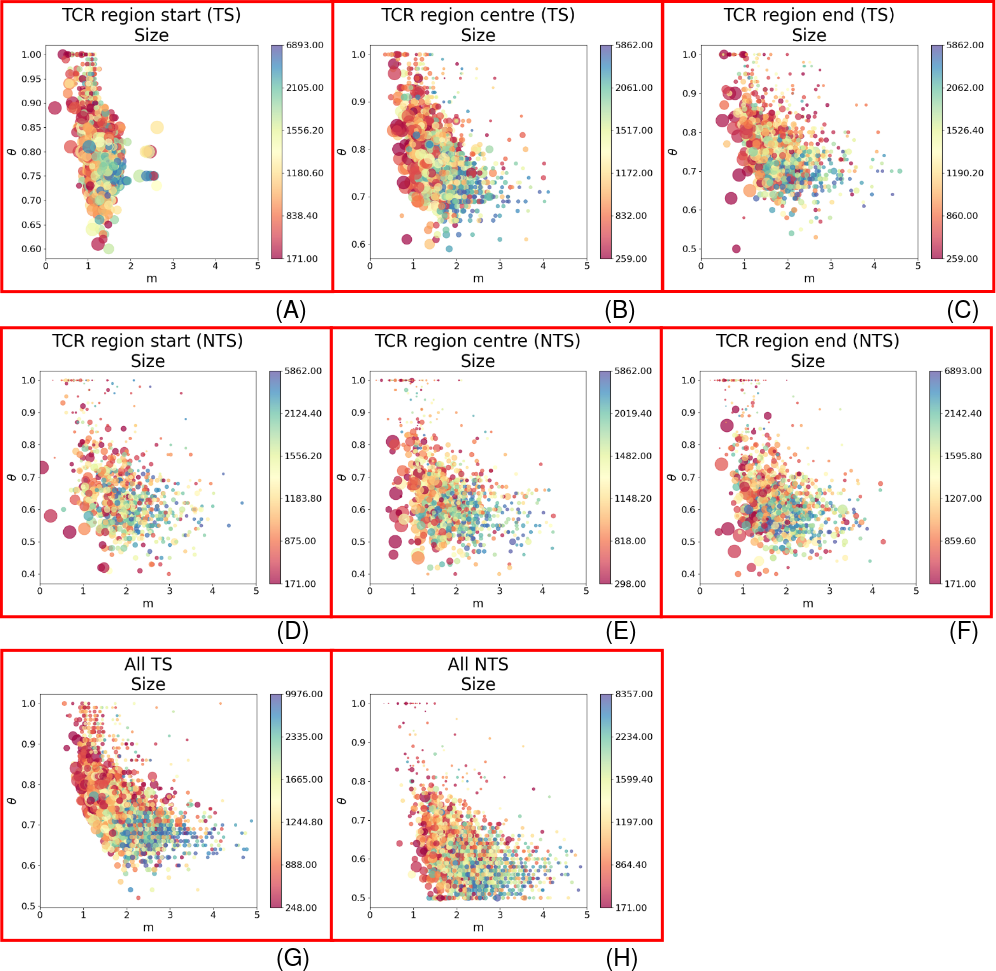

Supplement: S9 Fig — The size is clearly influencing repair for both, TS and NTS in the TCR and gene configuration. The x and y-axis give the values of m and θ, respectively. The size of the circles show 1/τ: the larger the circle, the shorter the characteristic time. Significant interrelationships are marked with a red frame. (TIF) [file pcbi.1010488.s021.tif]

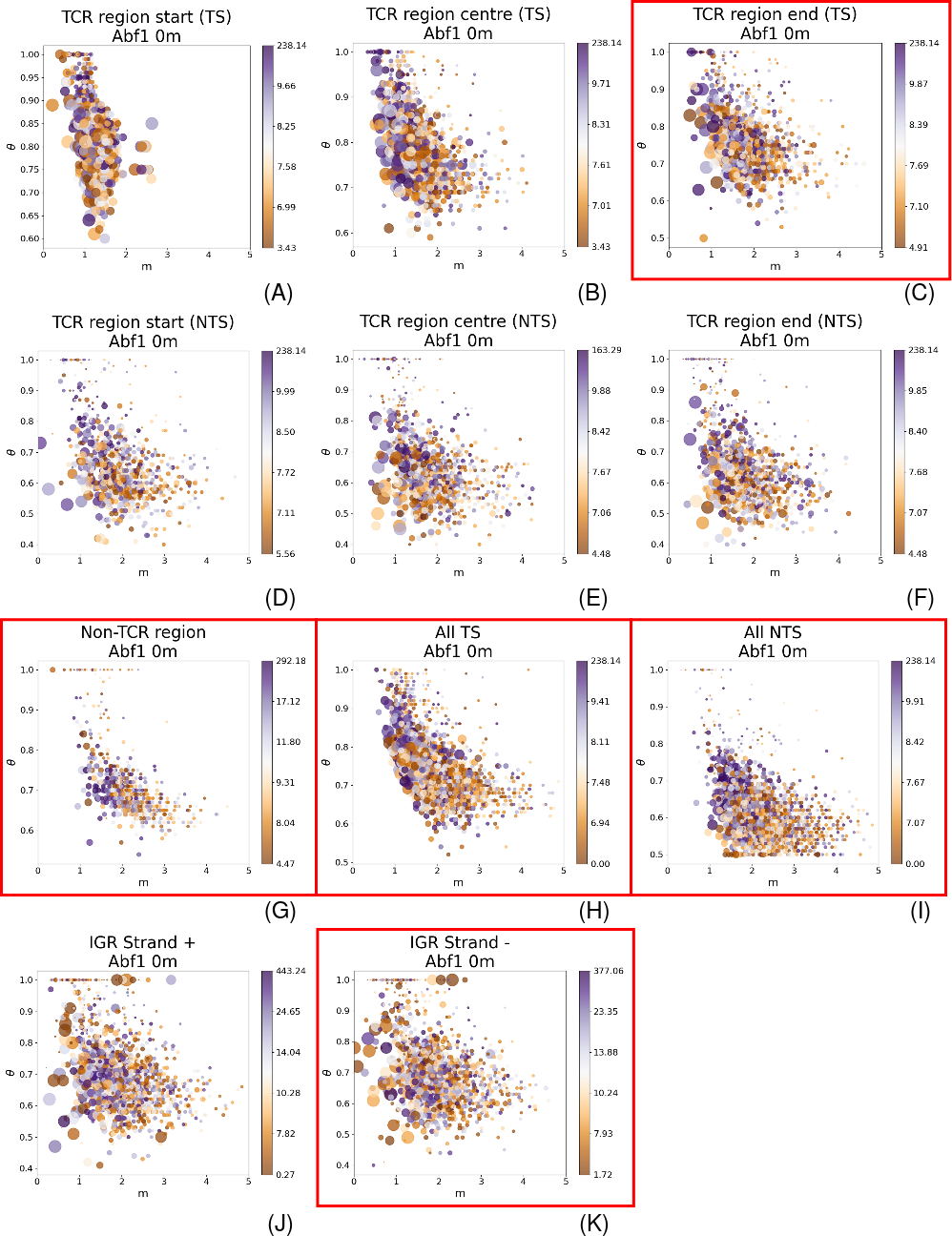

Supplement: S10 Fig — The results for Abf1 are more ambiguous. Although we can find a significant correlation to non-TCR regions as expected in the TCR setup, the picture is less clear for the gene configuration. The x and y-axis give the values of m and θ, respectively. The size of the circles show 1/τ: the larger the circle, the shorter the characteristic time. Significant interrelationships are marked with a red frame. (TIF) [file pcbi.1010488.s022.tif]

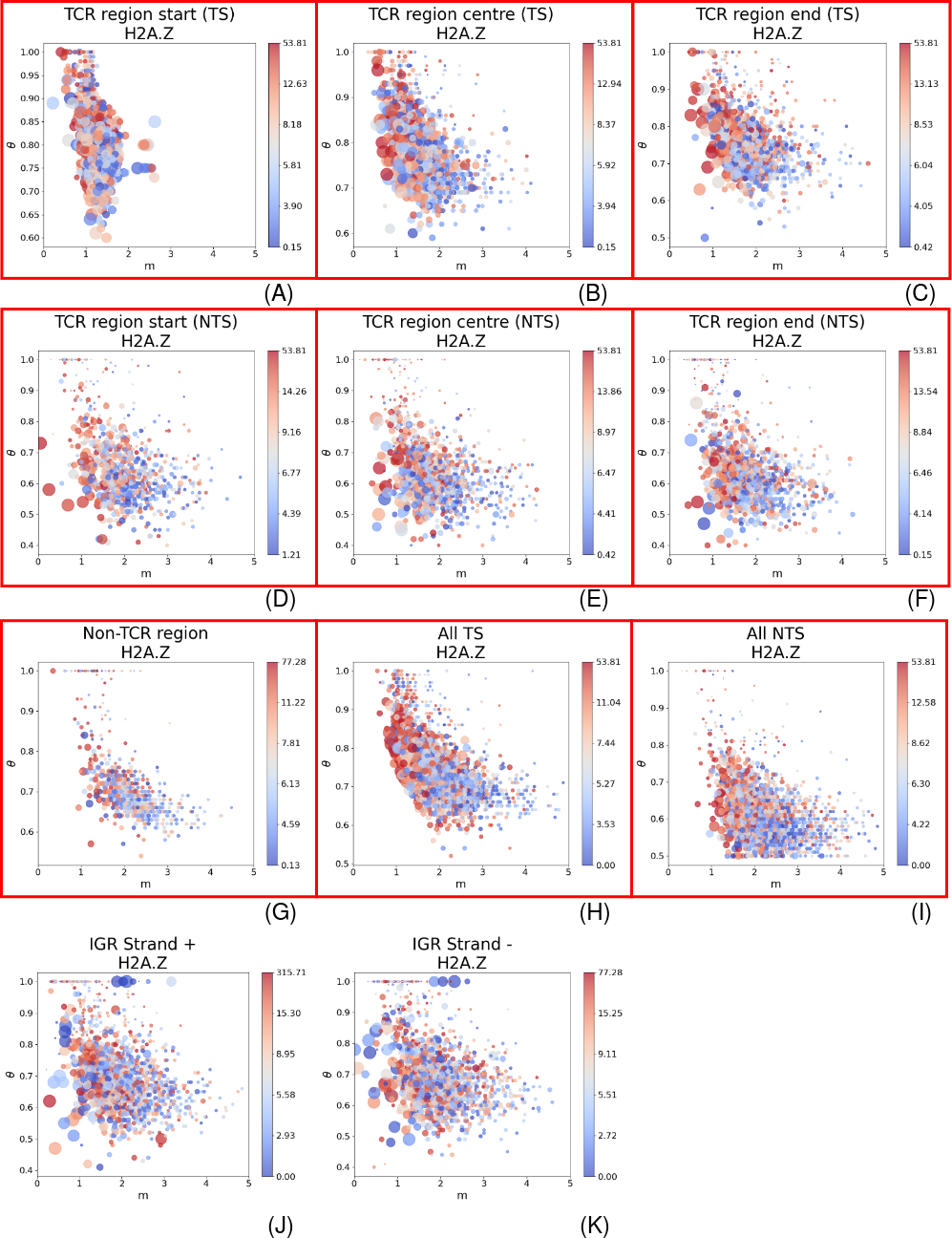

Supplement: S11 Fig — Similar to Abf1, the correlations with H2A.Z do not allow a straightforward interpretation. Whilst repair in all areas in the TCR configuration is seemingly linked to H2A.Z, this tends to be restricted to the TS and NTS in the gene setup. The x and y-axis give the values of m and θ, respectively. The size of the circles show 1/τ: the larger the circle, the shorter the characteristic time. Significant interrelationships are marked with a red frame. (TIF) [file pcbi.1010488.s023.tif]

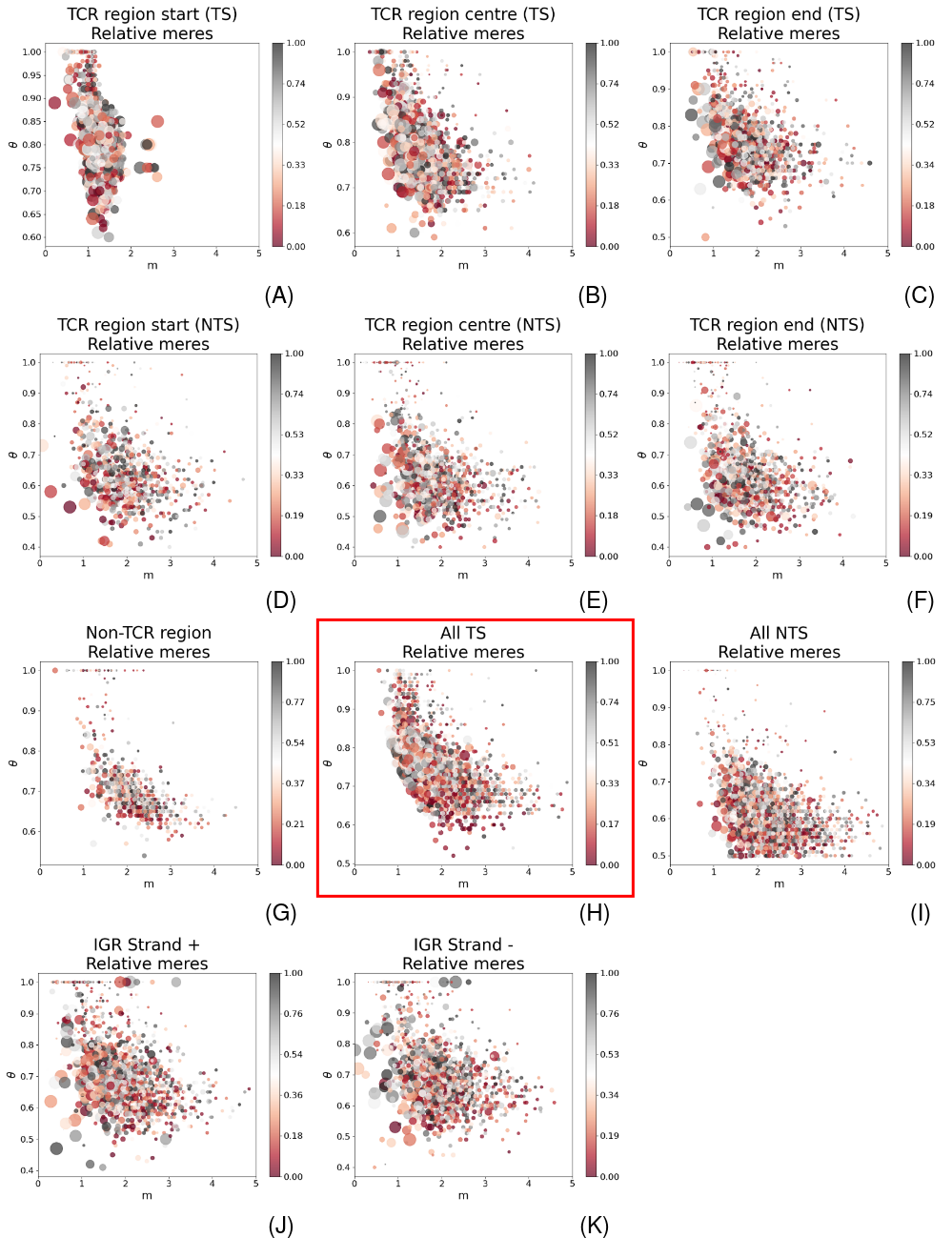

Supplement: S12 Fig — With the exception of the TS in the gene setup, the distance to telomeres or centromeres (shortened with meres) does not affect repair dynamics. The x and y-axis give the values of m and θ, respectively. The size of the circles show 1/τ: the larger the circle, the shorter the characteristic time. Significant interrelationships are marked with a red frame. (TIF) [file pcbi.1010488.s024.tif]

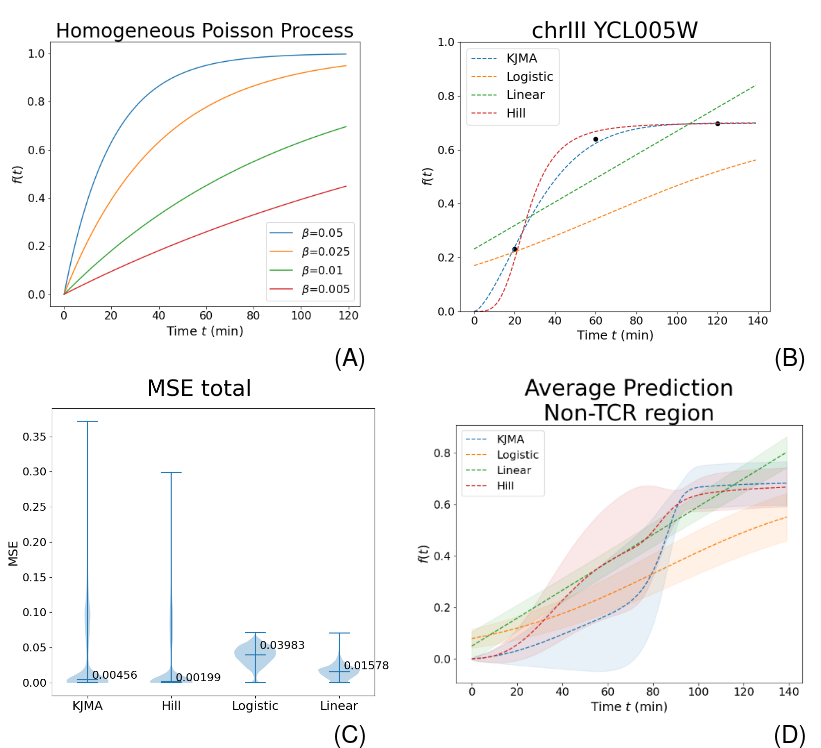

Supplement: S13 Fig — (A) A homogeneous Poisson repair process with λ(t) = c has the strongest change in the beginning which subsequently flattens out. In most investigated regions, such a behaviour is not observed. (B) We compared the performance of different models to describe the data, which is exemplified for gene LDB16 (YCL005W). Black dots represent the repair data (converted CPD-seq data, see Eq 7), whereas the best fit of each model is given in dashed lines. (C) We applied the mean-squared error (MSE, S7 Appendix Eq 1) to compare the performance of the models with respect to the data. The KJMA model and the Hill equation perform undoubtedly better than simpler models like linear or logistic regression. Nevertheless, the Hill equation describes the data slightly yet significantly better. It should be emphasised though that the performance difference is marginal. Width of the shaded areas represents the number of genes that yielded the corresponding error, which is mirrored at the vertical line. The centre horizontal line with the corresponding numbers give the error median. The top and bottom horizontal lines show maximum and minimum, respectively. (D) Despite the fact that non-TCR regions are not expected to show an observable impact by TCR, the Hill equation indicates two mechanisms that act at different time points. Dashed lines give the mean whereas the shaded areas show the standard deviation. Together with the fact that there is no straightforward interpretation of the Hill equation in context of repair evolution, we conclude that Eq 3 is a sensible choice. (TIF) [file pcbi.1010488.s025.tif]

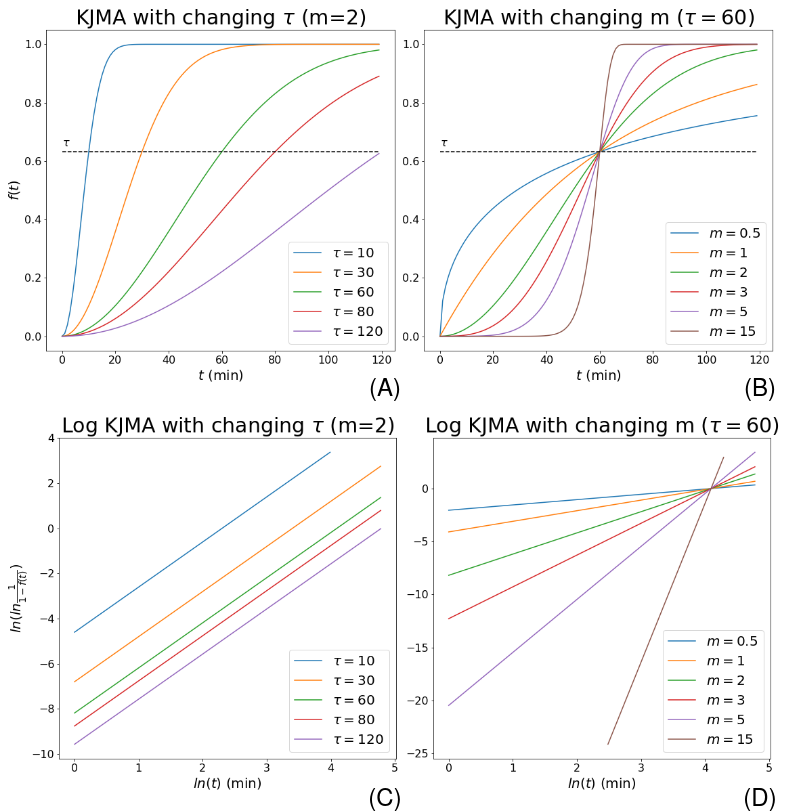

Supplement: S14 Fig — The KJMA model includes two governing parameters (as the original model does not involve θ), which are exemplified in (A) for m and (B) for τ. Eq 3 can be conviniently converted to a linear regression problem which is shown for the parameter settings of (A) in (C) and for the parameters of (B) in (D). (TIF) [file pcbi.1010488.s026.tif]

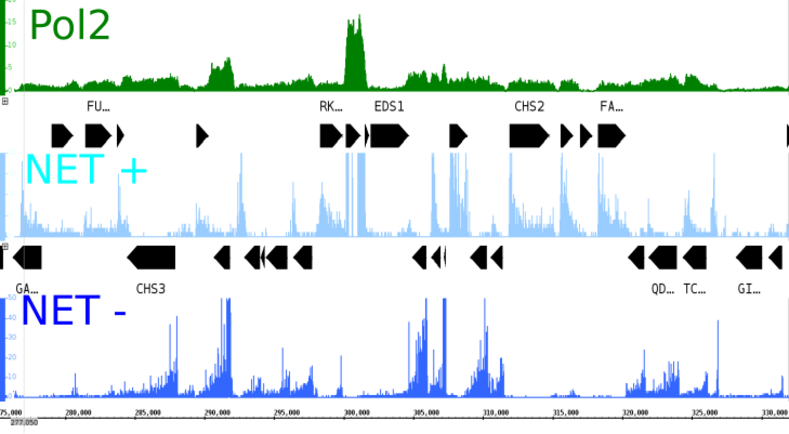

Supplement: S15 Fig — The example of the NET-seq signal in comparison to the Pol2 ChIP-seq data probed by [41] shows that Pol2 exhibits a constant augmentation of the signal amplitude at transcribed regions, whereas NET-seq data decrease as a function of distance from the TSS. The Pol2 data is coloured in green, whereas NET-seq is given in blue (light blue represents the Watson, and dark blue is the Crick strand). The example is given for chromosome II around CHS2 and CHS3. (TIF) [file pcbi.1010488.s027.tif]

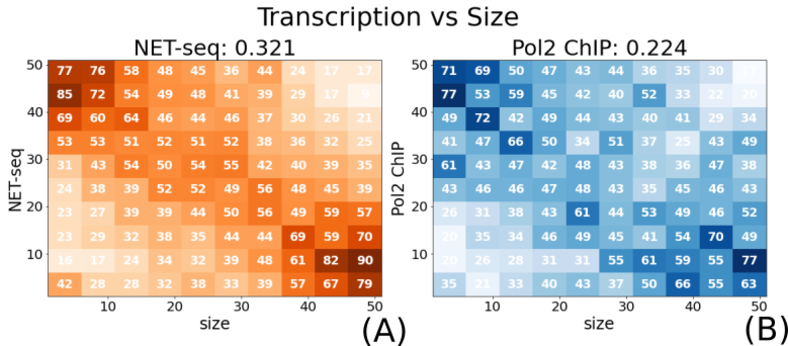

Supplement: S16 Fig — The plots show the two-dimensional histogram distribution of TU length and different measurements of transcription rate. The number of genes per bin is given through the colour intensities and white numbers. The DC (S4 Appendix Eq 3) per measurement is given in the title. Size and transcription rate data was divided into 50 equally sized bins, which is given by the x and y axis. We use the 95th percentile to remove strong outliers. (A) The histogram distribution of NET-seq transcription with respect to size reveals that smaller genes tend to have higher transcription rates than larger genes. (B) This link is weakened when considering Pol2 ChIP-seq data. (TIF) [file pcbi.1010488.s028.tif]

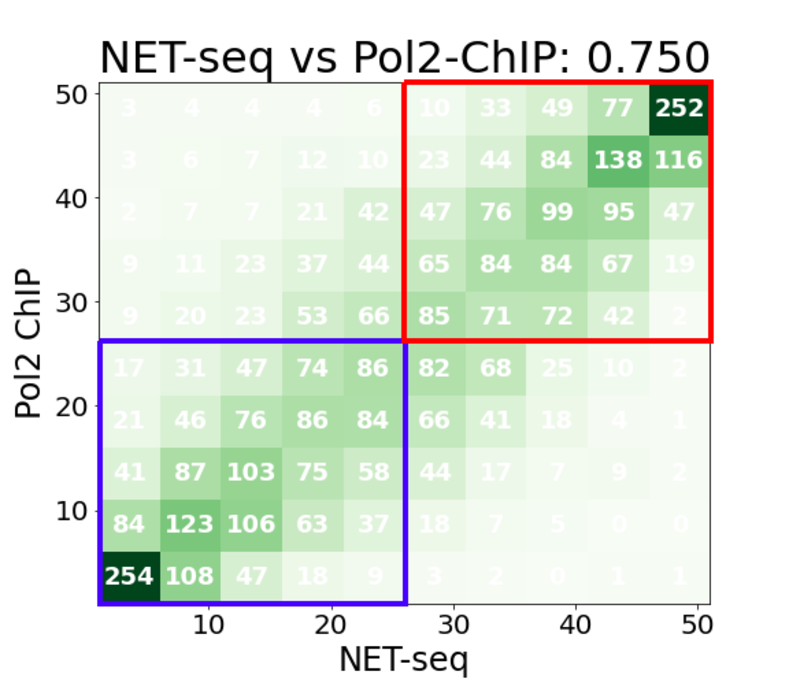

Supplement: S17 Fig — The plot shows the two-dimensional histogram distribution of NET-seq and Pol2 data. The number of genes per bin is given through the colour intensities and white numbers. Size and transcription rate data was divided into 50 equally sized bins, which is given by the x and y axis. NET-seq data and Pol2 ChIP-seq signal are strongly related (DC = 0.75, S4 Appendix Eq 3). As we consider only two groups of genes with respect to transcription, i.e. genes with a low (blue) or high transcription rate (red), we can confirm that the majority of regions fall into the same category. (TIF) [file pcbi.1010488.s029.tif]

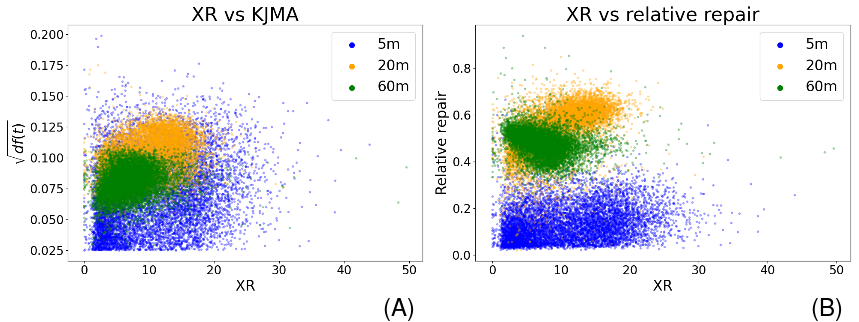

Supplement: S18 Fig — (A) The model predictions in the gene configuration are less correlated with the XR-seq data than in the TCR setup (DC = 0.241, S4 Appendix Eq 3). (B) When correlating the relative repair rate and the XR-seq data in the gene setup, the DC is as low as for the model predictions (DC = 0.231). Therefore, we assume that the weak linkage is due to the data segmentation. (TIF) [file pcbi.1010488.s030.tif]
