# Supplementary material for: A quantitative modelling approach for DNA repair on a population scale
Source: PLoS Comput Biol. 2022 Sep 12;18(9):e1010488. doi: 10.1371/journal.pcbi.1010488 (PMC9499311; doi:10.1371/journal.pcbi.1010488)
Supplement: Attachment — Submitted filename: Zeitler_2022_Response to Reviewers_200622.pdf [file pcbi.1010488.s031.pdf]

Point-by-point response to reviewers' comments on the manuscript by Zeitler *et al.* (PCOMPBIOL-D22-00499)

Introduction and general response to the main concerns about the KJMA Model

We thank both reviewers for their constructive and valuable feedback, which helped us to significantly improve readability and accessibility of our manuscript for a wider audience. We agree that some motivational aspects that were aimed to justify the applicability of the KJMA model do seem far-fetched from a pure biological standpoint. We incorporated the proposed suggestions to substantially revise and restructure the presentation of our results. In the following, we first summarise the changes to address the reviewers' main concerns and subsequently provide a detailed point-by-point answer for each comment.

Initially, our derivation of the KJMA model equation—which is the central point of our study—was motivated by stochastic Brownian motions of particles in the nucleus. We aimed to model the diffusion-search behaviour of repair proteins to find CPD damage. This was coupled with another random process which symbolised repair time or accessibility to the lesion. We were amazed to find that this equation resembles the KJMA equation to model phase transitions in solids. We hoped that the abstract angle could initiate other researchers to re-think their current computational pipelines and develop other tools to exploit multidimensional properties of the data (i.e. cells and genome positions). Since we are convinced that the understanding of sequencing data representing an average cell can be flawed, we made this the pivotal point of our previous version. The physical comparison was intended to make it rather independent from the actual area of application. However, reading the comments made us realise that the emphasis on the KJMA model removed focus from the three main aspects of our work: the motivation that a computational framework is needed to recover missing information from sparse sequencing time points; the understanding of the data which should be multidimensional; and the actual findings that the analysis pipeline produces.

Consequently, we shifted the paper's storyline back to the initial motivation of the equation. Proteins diffuse through the nucleus and subsequently repair CPDs. Both of these processes are represented by a random variable. We hope that the biological motivation of the equation can attract a broader audience, as the underlying assumptions are less abstract and more applicable to actual cellular processes. We want to emphasise that this neither changes the algorithm nor the results. Understanding the data evolution as growth of abstract patterns—the previous focal point—is now significantly shortened and given as an alternative interpretation in the Discussion section (page 17*f*).

Reviewer #1: I wonder if there is a flaw in the analogy between the physical role of the KJMA model and how it is being superimposed on the biological pattern of repaired sites. I find the argument that underlies figure 1 to be problematic: the assumption that the repair is independent between cells and between sites within cells, which is required to gather the repaired sites into the contiguous block 'pattern', needs much more discussion and evidence. But even then, it is apparent that the spatial metric properties of the equation in the physical application of this equation means that in the domain for which it was derived, the KJMA equation makes a strong use of the fact that one can't freely permute the spatial coordinates arbitrarily. The paper as it stands doesn't really resolve this conflict - it's possible

that a major re-phrasing of the paper so that the KJMA model is entirely motivated by the biological setting would resolve this (it's unnecessary to mention the physicists' use of the equation in anything but the most fleeting way, if there is an inherently biological motivation for it), but the situation where phase regions can only grow on their boundary, for instance, doesn't have a clear translation to me when phrasing in terms of repair sites: after the 'pattern' is formed as in figure 1, why are some sites 'special' (appearing on the boundary) whilst others aren't.

Answer:

This is an important point. Independence between cells is an essential underlying assumption of the model. We substantially restructured the presentation of our modelling approach to resolve conflicts with an interpretation derived from physics (see also our response above to the main concerns). In the revised version, we constructed a motivation that does not require independence between repair processes in a single cell. This becomes only important when motivating the data evolution based on the KJMA model, which has been shortened to a subsection in Discussion (page 17*f*). An in-depth analysis of the applicability and consequences of the pure “KJMA-understanding” was moved to S8 Appendix.

We addressed the initial two independence assumptions (non-interfering repair between and within cells) in the previous version in Discussion (page 12*f*) (and partly in Introduction, page 3). They can be summarised as follows. We find the independence between cells trivial. A similar understanding underlay all sequencing data analyses that we are aware of. Commonly, the signal is explained in the context of an average cell, which implicitly assumes independence to make results comparable. If this were not the case, the exact same conditions must be replicated (e.g. spatial distance between colonies and cells) in each experiment to compare and validate results; and subsequently, any other presumption than independent repair between cells would diminish the significance of previous NGS results. We also conjectured independent repair within a single cell. We based our argument predominantly on a study which showed that there are only 0.4 to 0.5 CPDs per 10kb. The cited paper used a similar UV dose on *C. elegans* which we hope is sufficient to make the results applicable to budding yeast. Unfortunately, findings for *S. cerevisiae* were not available to our knowledge, but similar outcomes (small number of lesions over long genomic areas) were obtained by studies on human cells, although with a different UV treatment. Furthermore, research on Chromosomal Interaction Domains (CID) indicates that processes in different domains are fairly independent of each other. As CIDs are commonly smaller than 10kb in *S. cerevisiae* (2-5 genes; ~ 2 to max 10kb), we find it unlikely that there is more than one lesion per domain. We therefore assumed independence within a cell.

It is entirely possible that the KJMA model merely describes a stochastic point-process of repair, which seems a much simpler and more intuitive explanation, and that the time-profile of fraction of repaired sites is purely an emergent phenomenon: early on the proportion of repaired is small because of the lack of accumulated time in which to undergo repair relative to the number of damaged sites, and that as time progresses the accumulation increases but then starts to tail off as the number of available unrepaired damage sites becomes sparser. There is nothing inherently two-dimensional about this point-process approach, and if it

arrives at a similar or identical functional form for the time dependence of the repair rate, then the rather forced analogy could be removed.

Answer:

We thank Gavin Kelly for pointing out the stochastic nature of the repair process. This is absolutely correct. In the way the KJMA model is applied in the context of CPD removal, the growth of abstract repair patterns is a consequence of independent points (i.e. repaired positions) that are re-ordered. The presented equation describes indeed a random point process. We do think that a two-/multidimensional understanding of the data is important to appreciate that the transition between two following time points cannot be simply linearly interpolated (since it should represent independent cells). This is why we initially put focus on the KJMA model. However, we agree that the understanding from the perspective of a point process is much easier to follow. In the revised version, we set more emphasis on the biological process (now explained through a Poisson point process, see pages 5*f*) to motivate the grid representation of sequencing data.

The use of KNN in the correlation of parameter estimates to biological features seems overly and unnecessarily complex - a data-dependent binary split of the data (it's not clear if this is done on the parameter estimates or the biological features) and then doing a t-test seems prone to measurement error bias - a non-parametric test of association between the two continuous variables would seem more straightforward, or a spline regression if a non-linear association was suspected.

Answer:

We agree on the pivotal role that is allocated to the correlation analysis. It is indispensable to show that the used significance test does not measure any artefacts that are induced by the used index. We discussed this in the previous version in Results (Correlating Repair Dynamics With Biological Properties) and in Discussion (sections Correlating CPD Repair Dynamics With Genomic Properties and Parameter Optimisation and Definition of Correlation). However, we realised that some important aspects were not properly emphasised and set into context. In the revised version, we provide additional arguments in Methods and Materials (page 21*f*) and S5 Appendix. The main argument that speaks for the application of a *k*NN is the following: it poses barely any requirement to the input and output distribution, either of which can have any dimensionality. It can deal with large variances without assuming that they are insignificant and can be smoothed out. The paragraphs below motivate the approach in more detail.

Indeed, there are some common and straightforward nonparametric significance tests to find correlation in two multidimensional data sets, and we made use of various ones. One example is the distance correlation (DC). However, we need to deal with two particularities in sequencing data. Firstly, the data include presumably a substantial amount of variation or noise; and secondly, the data histogram distribution peaks sharply around a (usually low) value with a long tail for positive values. During our study, we compared different statistical models next to the *k*NN approach, i.e. a Gaussian process (GP) as an example for nonparametric regression and a linear regression model (although not published in the main text, see source code on GitHub). Considering the noise, we found it almost impossible to make significant predictions for values around the histogram peak. Unfortunately, this

represents a large part of the data signal. We therefore deemed GP and linear regression to be inappropriate. Classic indices such as Pearson's correlation, DC, or mutual information also indicated no or low correlation for any data set for the same reason. Instead of finding accurate predictions, we therefore implemented a classification approach to capture general trends (i.e. high versus low values). This, on the other hand, made the aforementioned indices inapplicable. For example, the distance between all points in the same class is zero, but the parameters exhibit variation, which renders the DC meaningless.

Spline regression implicitly assumes a slowly varying signal, which suggests that all high frequency changes are mere noise. Moreover, it requires the discretisation of the multidimensional input space (repair parameters) into ranges rather than the output (sequencing data). We initially deemed it to not correctly address the stated difficulties in sequencing data. However, following Gavin Kelly's suggestion, we added a correlation test with a linear spline regression as well. We tested 3, 5, and 10 ranges per parameter. When it was applied to a classification task, all regression coefficients for all correlations were substantially non-random. Yet the found mapping was far off from any reasonable correct predictions (Accompanying Fig 1 for reviewers below). We tried several numbers of classes (i.e. 2, 5, 20, 50) with an equal amount of data each to rule out any sensitivity bias. When output data was not discretised (regression instead of classification), no interrelationship was found to be significant. Here, we define an interrelationship to be significant when all parameters have a p-value lower than 0.1. We found it difficult to account for the right parameter ranges to establish sensible significance values whilst also having a reasonable representation of the functional relationship.

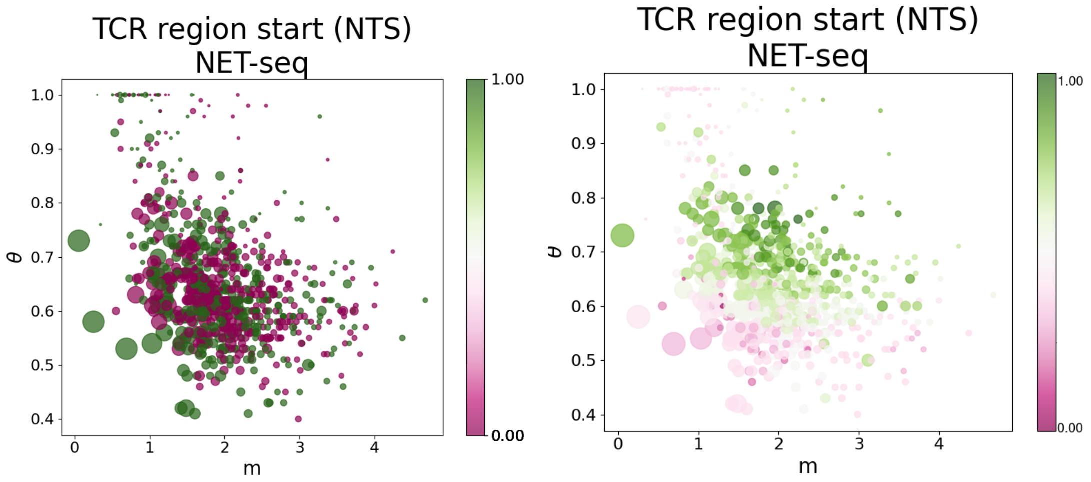

Accompanying Fig 1 for reviewers: The figures show the parameter distribution coloured with respect to NET-seq data. The x and y axis indicate the abnormality constant and maximum ratio of repaired cells, respectively. The circle size shows the scaling parameter β. Although spline regression indicates a significant link, the mapping does not seem to correctly represent the classification data (compare real distribution on the left with spline mapping on the right). When spline regression was applied to the actual sequencing data (no discretisation was applied), then no mapping was significant.

Instead, we aimed for a nonparametric classification approach to minimise the requirements for the distributions. The comparison with a random baseline model allowed us to detect a

decent interrelationship (in comparison with the baseline) and measure significance at the same time. The classification error also gives an indicator (though admittedly not strong) for the intensity of the established link. The *k*NN classification approach could easily deal with large variances and therefore outperformed the GP and linear regression model. Even more important: due to the fact it is nonparametric, we did not incorporate any initial bias about the nature of the mapping (in contrast to defining ranges for splines). It could hence easily account for correlations where one group is encircled by another (see previous Fig 7 that corresponds now to supplementary S6 Fig in revised version, where the red class is surrounded by blue values). These interrelationships are often difficult to capture with simple correlation indices. The model was trained with a randomly divided training data set and verified with a mutual exclusive test set. By repeating the procedure 100 times (which is far more than in common machine learning approaches that use *k*-fold cross-validation for example), we presume that a possible data-split dependent bias is removed. The t-test of the prediction error allows furthermore to detach particularities of the mapping (i.e. *k*NN, GP, and linear regression) and makes the results of the different models comparable (independent of whether parametric or nonparametric). We find that the implemented algorithm was necessary to account for all the mentioned challenges and to generalise obtained results.

I found the impact of the concerns about the applicability of the KJMA model overshadowed my reading of the rest of the paper. The testing of association between biological features and repair rates would have been more convincing if it had been done directly (perhaps empirically, even a seriation or simple PCA decomposition of the signal clustering being graphically annotated with the biological feature) in addition to going via the correlation with KJMA model parameters.

Answer:

Indeed, we aimed for a data-driven / empirical correlation approach (see previous point). We re-structured the paper to present it more coherently. A PCA decomposition of biological features with respect to parameters was given previously in Fig 7 and has been moved to the appendix S6 Fig.

Thank you for supplying the github code - I would recommend a DOI service so that a more permanent record can be associated with the code.

Answer:

Many thanks to Gavin Kelly for this suggestion. We were previously not aware of this possibility and have added a DOI to the repository.

Reviewer #2: The authors consider a quantitative treatment of the dynamics of DNA repair via an application of the KJMA model. Parameters of the model are tested for correlation with various biological quantities such as transcription, finding a novel connection between repair dynamics and gene size.

Some aspects of the approach and results are, to the best of my knowledge, novel. Moreover, I believe that the findings presented will be of interest to the field. However, I do have some concerns regarding interpretation and presentation of the work.

Major Concerns:

1. Connection to the KJMA model

I actually find the mapping of DNA repair to the KJMA model a bit of a stretch. The authors assume (reasonably) independence of repair within and between cells. They also claim that this allows DNA repair to be modelled using the KJMA model as new regions of repair can be rearranged to give a growing pattern on a two-dimensional grid. However, it seems that independence really means that DNA repair should be modelled as independent nucleation events that do not grow and much of the connection to KJMA model would seem to be lost. The authors should either strengthen the justification for the use of the KJMA model or remove some of the emphasis on its use from the manuscript. If the assumption is that nucleation is time dependent such that an S-shaped curve is expected for the progression of repair, then, in the absence of any further information, any simple model that produces an S-shaped response should be applicable. It might be reasonable to suggest employing the KJMA model as it is well known and produces an appropriately shaped response curve, or perhaps the authors can show that the KJMA model is capable of treating the case of time-dependent nucleation without growth.

Answer:

We agree that the important aspect of the KJMA model is its S-shaped curve. We thank Reviewer #2 for pointing out this critical characteristic. We model DNA repair as an irreversible process from *damaged* to *repaired*. The probability distribution for the transition over time must hence be described by a monotonously increasing function between 0 and 1. An S-shaped trajectory whose derivative is asymmetric around the inflection point gives the largest flexibility for a comparatively simple model. A corresponding paragraph has been added to the Discussion (page 15). Moreover, we significantly revised the structure of the paper to explain CPD repair from a more biological viewpoint (see reply to main concerns above). This should remove the concerns about the applicability of the KJMA model per se. However, the growth of abstract repair patterns is a crucial aspect of the model, and nucleation alone is not sufficient. This was elaborated in Discussion of the previous version (page 12*f*), where we described the meaning of the nucleation rate n and growth speed G. In the following, we refer to the physical interpretation. The transformed volume from a nucleation site within time t which is driven by G is linked to the dimensionality of the space and therefore includes the shape parameter m. On the other hand, n is assumed to be constant. If repair were represented solely by n, the equation would follow the form $f(t) = 1 - \exp(kn)$ where k is a constant scaling parameter. Such a function has the strongest increase in the beginning and subsequently abates. This can be understood as repair being only dependent on the remaining number of lesions. We find that this cannot accurately represent the data evolution over time. We added a supplementary section S7 Appendix to compare the KJMA with other models. We addressed the concern regarding a constant repair rate (nucleation rate without growth) in Discussion (page 14) with respect to the biological

interpretation. Consequences of a constant/independent nucleation rate from a physical perspective are discussed in S8 Appendix.

2. Small number of time points

The authors do acknowledge that an important future step is to apply the modelling approach to data with greater time resolution. Here, they apply a three-parameter model to a time course with only four points. Arguably, they have a sizeable data set in that the data is genome wide, covering many regions of repair which can be partitioned into distinct types. However, this is quite a low number of points for each individual fit. I would recommend that the authors provide some additional analysis on model selection, ideally showing that the S-shape curve provides a significantly better fit to the data than the simplest alternative (which would be a linear model). Alternatively, the correlation results could be compared to a similar approach with a linear model, ideally showing that the approach with an S-shape curve is more powerful in revealing links between the chosen biological quantities and the dynamics of repair.

Answer:

We thank Reviewer #2 for emphasising this crucial aspect, as it shows that we have not sufficiently highlighted it. We definitely acknowledge that the number of time points per data set is low. From our point of view, this is the most important reason why it is necessary to have an analysis pipeline to recover missing transitions. The model is applied after the data was created (i.e. to already published data). It was designed to extract additional information about missing dynamics and to make them hence comparable to future studies with different techniques or narrower time points. This aspect was put more into focus in the revised version. We also show in S7 Appendix and S13 Fig (revised version) a comparison with other models, i.e.one linear and three S-shaped functions. As shown in S13 Fig, the KJMA function and Hill equation clearly outperform the logistic and linear regression model. However, the Hill equation seems to be slightly but significantly better. We show in S7 Appendix that the Hill equation can be understood as a first order approximation of the KJMA model with slightly larger flexibility. This allows a better fit to the data. Nevertheless, the Hill equation describes quantities rather than time and cannot be straightforwardly interpreted with respect to temporal data. We find therefore the choice of the KJMA model to be reasonable.

3. Potential problem with the fitting algorithm

The authors claim that for data showing no (or negative) repair levels at 20 minutes obtaining good fits is difficult (Figure S2B and associated discussion). I can estimate what seems to be a superior fit to YAL048C with values of theta = 0.7, m = 6, tau = 45 (all within the allowed ranges). The authors should provide more reasoning as to why the apparently poor fit in Figure S2B was chosen by the algorithm.

Answer:

We thank Reviewer #2 for raising this important point. We re-analysed our entire source code. Whilst the fitting algorithm was shown to be correct, we found indeed a bug in the algorithm that evaluates the significance of a potential link to other genomic data. We

corrected the error and changed a few values in S3 Table as well as adapted the red frame around the plots that indicated correlation. We are relieved to announce that no major result was jeopardised. Regarding the parameter estimation, it is undoubtedly true that several values could obtain reasonable results. However, the transformation to a linear regression problem constrains the parameters to unique values. This is exemplified in the figures below as well as in S3 Fig of the revised version. The parameters given by Reviewer #2 are indeed seemingly a better fit in the normal *time-repair fraction* plot (Accompanying Fig 2 below). However, our parameters are optimal when transforming the problem according to Eq (2) of the previous version (which corresponds to Eq (5) in the revised version) (Accompanying Fig 3 below). This is true for both θ values, the one given by Reviewer #2 (Accompanying Fig 3 below left) and the θ determined by our algorithm (Accompanying Fig 3 below right). We want to highlight that the red line is much closer to the data point at 60 minutes. This demonstrates that we can find other representations by biassing the fitting procedure towards certain time points. Nevertheless, we wanted to impose as few constraints as possible.

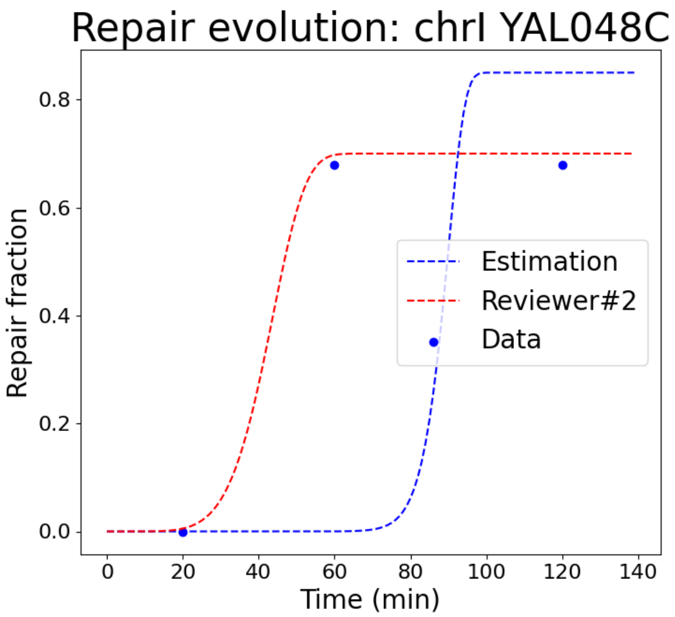

Accompanying Fig 2 for reviewers: The trajectory given by Reviewer #2 (red dashed line) approximates the data (blue dots) better than the found parameters (blue dashed line).

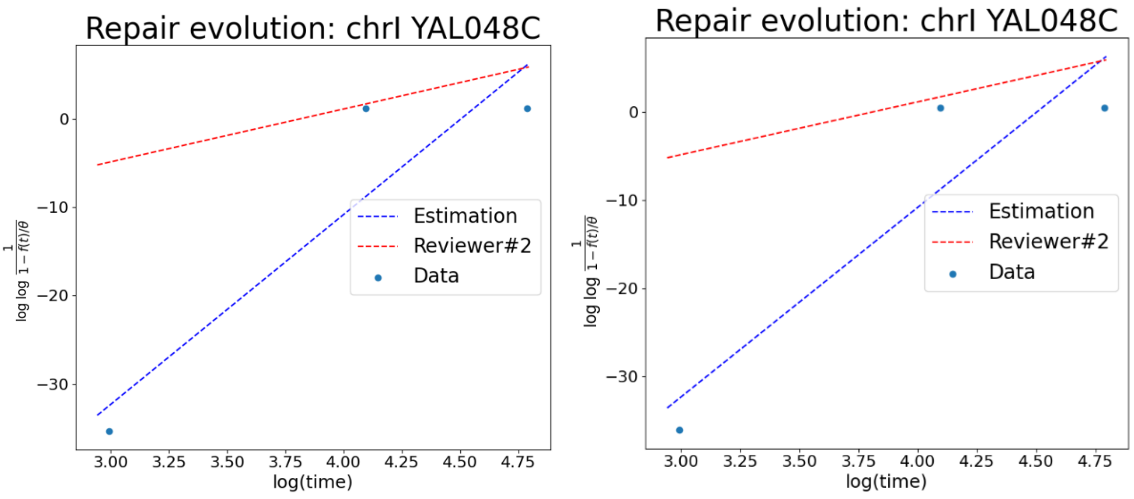

Accompanying Fig 3 for reviewers: When solving the linear regression problem in the double-log converted space, the parameters found by the algorithm (blue dashed line) approximates the given data points (blue circles) more accurately than the parameters suggested by Reviewer #2. This is true independent of θ. Left: space converted with respect to θ proposed by Reviewer #2. Right: Converted space according to θ proposed by our algorithm.

4. Overall presentation

The manuscript was quite difficult to follow in places, often putting heavy emphasis on minor details and giving limited room to what I considered to be important points. I recommend some streamlining of the manuscript to improve readability.

Answer:

This is a very important point, and we thank Reviewer #2 for raising it. Our initial intention was to make the abstract repair space as intuitive as possible while deriving the formulas, but we understand that this might have been difficult to follow. With the major revision, we developed a different storyline which should be more intuitive (see above our reply to main concerns).

Minor Concerns:

5. The authors state that TCR is not expected to function on the non-transcribed strand. Has any potential repair coupled to non-coding antisense transcription been considered here?

Answer:

Antisense transcription has not been explicitly considered. However, we presume that its impact is negligible. This is motivated by the fact that highest transcription rates in yeast occur on the transcribed strand (TS) of protein-coding genes. However, there is a considerable number of TS that showed a large transcription rate but slow repair, which were classified as non-TCR. We hence do not assume that there is a sufficiently strong link to consider it to be detectable in the used CPD-seq data. Future studies will be needed to

directly address the relationship between non-coding antisense transcription and repair rate by specifically designed experiments.

6. Figure 2 – the top and bottom panels appear to have been switched compared with what is described in the main text and the figure caption.

Answer:

Following the reviewer's comment, we changed this in the new version (S14 Fig).

7. Some of the description in the main text is more suited for the methods section and some of the description in the methods section might be more suited for the main text.

Answer:

This should be resolved through the major rephrasing of the new version. The interpretation from a physical perspective has been moved to the Discussion. Explanation of the data segmentation and details about the significance are now in the Methods and Materials.

8. Figure 6 is missing a caption title (all other main-text figures have this).

Answer:

We did indeed forget to format the caption in bold (although it was there). Thanks for pointing that out, we changed it in the new version (Fig 4).

9. Figure 4 – the legend spots were too small for me to easily distinguish between the colours.

Answer:

We updated the figure in the new version to include larger spots in the legend (Fig 2 and S18 Fig).

10. Figure 3 – the colour scheme is confusing: I don't think blue and orange should be reused for the lines at the bottom as they appear to represent something different there.

Answer:

Following Reviewer #2's suggestion, we used a new colour scheme in the revised version (S1 Fig).

11. Figure 3 – I think it would be helpful to also describe additional classifications in this schematic, including TS, NTS, beginning, centre and end.

Answer:

We marked the beginning, centre and end of the TS in a new version of the figure (which was moved to S1 Fig in revised version).

12. Figure 5 – the meaning of the lines and shaded areas should be in the figure caption. How the averages were taken should also be described in sufficient detail somewhere in the manuscript.

Answer:

This has been updated in the new version (Fig 3).

13. Figure 5 (and connected discussion) – Is there some analysis that could be done in order to separate out early- and late- repair subpopulations (assuming that they are indeed what is driving the double-peak behaviour in the derivative).

Answer:

This is absolutely possible. A simple filtering algorithm could easily assign transcripts to the respective subgroups. However, as we did only intend to show that the model is able to recover two distinct mechanisms without explicitly including them, we did not perform a subsequent analysis.

14. References appear not to have been ordered.

Answer:

References were sorted in alphabetical order rather than with respect to its appearance. However, to make it clearer we changed the citation style to Name (Year) rather than using numbers.

15. Line 180 – “transcripts that exhibit strong repair …” – should this be transcribed regions as the transcripts themselves are not repaired?

Answer:

This is correct, and we corrected the sentence in the revised version.

16. Many of the supplementary figure captions are missing descriptions (for example, the meaning of the size of the circle points in the plots).

Answer:

We agree that this could further help the reading flow when the parameter plots are described again. We added the explanation to each plot (S7 Fig - S12 Fig).
