# Supplementary material for: A quantitative modelling approach for DNA repair on a population scale
Source: PLoS Comput Biol. 2022 Sep 12;18(9):e1010488. doi: 10.1371/journal.pcbi.1010488 (PMC9499311; doi:10.1371/journal.pcbi.1010488)
Supplement: Attachment — Submitted filename: Zeitler_ResponseToReviewers_Revision2.pdf [file pcbi.1010488.s032.pdf]

Point-by-point response to reviewers' comments on the manuscript by Zeitler *et al.* (PCOMPBIOL-D-22-00499R1)

Once again, we thank reviewers and editorial board for their feedback and comments, and we are delighted to see that our revised manuscript substantially improved readability and accessibility of the approach. In the following, we address all remaining concerns and recommendations step by step.

Reviewer #2:

I still think there might be issues with fitting equally to a data point which has been set to (or close to) zero due to the measurement being negative (an impossibility in the model). Such points could have larger errors on them than positive measurements. Perhaps a fitting algorithm that takes differences in errors into account would improve matters and allow more genes to be considered for further analysis. I appreciate that this would entail significant extra effort and be beyond the scope of this manuscript, but it is something that could potentially be covered in future work.

Answer:

We acknowledge that it is—to some degree—dissatisfying to remove some genomic areas due to their fitted parameters. Reviewer #2 pointed out correctly that this stems almost exclusively from the fact that these regions contain rectified data values, i.e. the repair value at 20 minutes was corrected to 0, or the data at 120 minutes was increased to the previous repair state (in the following, these areas are also referred to as *difficult regions*). We evaluated an amended fitting procedure that encompassed almost all data points. However, by including regions which possess a supposedly large error margin in the data, the conclusions were less reliable. As we have only three time points per area available, a precise analysis of the present variability is impossible. We are convinced that a more detailed CPD-seq time course is necessary to correctly treat difficult regions. In the following, we present first a high level overview over the problem, which is followed by a detailed analysis.

Data rectification is only applied if CPD levels rise over the time course, which we suppose to be a result of large variability in the corresponding regions. These corrections lead to the approximation of switch-like functions. However, this property plays an important role in our study. As we presume that regions with such a behaviour are not repaired by TCR—due to the lack of efficient early repair—they indicate the working of another pathway which we suppose to be GGR. Parameter values that lead to such a switch-like function allow a clear separation of these regions in parameter space. Subsequently, the mutual effect of TCR and GGR becomes clearly discernible, and we recover two temporally distinct mechanisms. Nevertheless, due to the evidently large variability in the data around these regions, precise predictions and correlation measurements with other processes are not possible. We therefore prefer to exclude these regions from the downstream analysis. We realised that it was indeed incorrectly stated in the manuscript that regions outside the set parameter range were removed for the entire pipeline. We want to highlight again that they were kept for

recovering TCR and GGR as well as for the comparison with XR-seq data. The sentence that mentions the exclusion in lines 150-152 was moved to line 199-201. We also moved lines 413-416 to the subsection Correlating Repair Dynamics to Genomic Contexts. Moreover, we provide some additional explanation why these areas were removed in S2 Appendix. The results in the manuscript were compared with an adapted approach for which we amended the data preprocessing and the fitting procedure as follows.

It is easy to change the algorithm to include these regions by enforcing at least 1%-point repair between two consecutive time points. The process is therefore seen as *strictly* monotonically increasing repair as a function of time. This avoids the issue of fitting rectified data that was set to zero, which was correctly pointed out by Reviewer #2. It has a negligible effect on the absolute repair values, but it substantially changed data point positions in log space (see Fig 1 for Reviewers, left). Moreover, we can model our confidence in the data accuracy by applying a weighted linear regression, where the weight determines the impact of the time point on the parameter estimation. After a coarse parameter search, we used for difficult regions the weights 1, 3, and 2 for the data points at 20, 60, and 120 minutes, respectively. Otherwise, all weights were set to 1. Rectified time points of an area can be therefore kept as a constraint whilst decreasing their influence. This leads to reasonable parameter fits for all regions that contain CPDs and exhibit repair at 20, 60 and 120 minutes (see Table 1 for Reviewers in comparison with S1 Table in the manuscript). An example for different regions is given in Fig 1 for Reviewers, right. The points and the dashed line in blue represent the difficult region *GEM1* that was reported in the manuscript (S3 Fig). The amendment to the algorithm indeed removes the step-like behaviour. Reasonable estimations of repair could still be found with parameters slightly outside of the ranges reported in the manuscript. In the given example here, we allow $m \in [0, 6]$ and $\tau \in [2, 1000]$. The increase especially for τ can be motivated by the fact that we only excluded areas with constant CPD levels from 20 to 120 minutes (or with no repair at all). The characteristic time for corresponding regions is commonly significantly larger.

Whilst the parameter fits are indeed convincing, they create additional clusters in the accepted parameter space over all areas of a region type (see Fig 2 for Reviewers in comparison with Fig4 (E) in the manuscript). This stems from the fact that all newly included regions contain the same bias in the data preprocessing (i.e. 1%-point) and apply subsequently the same weights. Unfortunately, we do not have additional data available to induce more variability. The previously clearly separated genomic areas with a switch-like behaviour were now brought closely to the regions with reliable data whilst still being strongly grouped together. Consequently, it becomes difficult to distinguish between their respective effects. While we are confident to claim that difficult regions cannot be repaired by TCR due to their variability and lack of early repair, we are much less certain about the reliability of the actual data points. Yet by including them, the distinguishable effect of TCR and GGR vanishes. Whereas there were previously two clearly discernible bumps in the function derivative, the TS centre now indicates a working of two mechanisms only through a slight variation in the standard deviation (Fig 3 for Reviewers, left). We want to stress this represents the same phenomenon of different dynamics acting at the same region. This can be better seen for the TS end where the two peaks become again better distinguishable (Fig 3 for Reviewers, right). As the parameters are now distributed in a similar range, reliable and unreliable areas create similar functions. Additional variability is only induced by the large variance of θ which create the bumps in the derivative. Nevertheless, this can only be made

visible through the standard deviation and not by the mean. Bringing these clusters closely together in parameter space makes a subsequent analysis of repair dynamics and influencing processes difficult. In our opinion, it is crucial to include more detailed temporal data for making reliable conclusions for these difficult regions. It is hence an issue of the data acquisition rather than the algorithm itself.

Nonetheless, we re-performed the correlation analysis with other nuclear processes for the amended algorithm. We are relieved to announce that no major result was influenced. Although the number of *k*NN models that found a non-trivial interrelationship changed sometimes, most conclusions about the significance did not change for the *TCR* setup. However, some of the outcomes for Abf1, H2A.Z, and relative distance to centromeres or telomeres in the *gene* configuration were impacted. As they were already non-conclusive before, we do not see any study finding jeopardised. A full overview is given in Table 2 for Reviewers. Since the previous algorithm delivered comparable results for finding repair influencing factors in the *TCR* configuration while making TCR and GGR clearly discernible, we find it to be preferable.

	**Region Type**	**Total #Regions**	**Kept #Region**
***Gene* setup**	TS	4973	4973
	NTS	4973	4973
	IGR +	4067	4027
	IGR -	4067	3998
***TCR* setup**	TS Start	1878	1877
	TS Centre	1878	1878
	TS End	1878	1878
	NTS Start	1878	1877
	NTS Centre	1878	1876
	NTS End	1878	1878
	Non-TCR	1763	1760

Table 1 for Reviewers. The number of models per region, before and after applying the requirements for parameter ranges. IGR abbreviates intergenic regions. Almost all regions can be reasonably approximated using weighted linear regression and strictly increasing repair. Regions that were excluded did either not include two adjacent pyrimidine nucleotides in their sequence or did not exhibit any change of repair between 20 and 120 minutes, which we gauge to be unlikely.

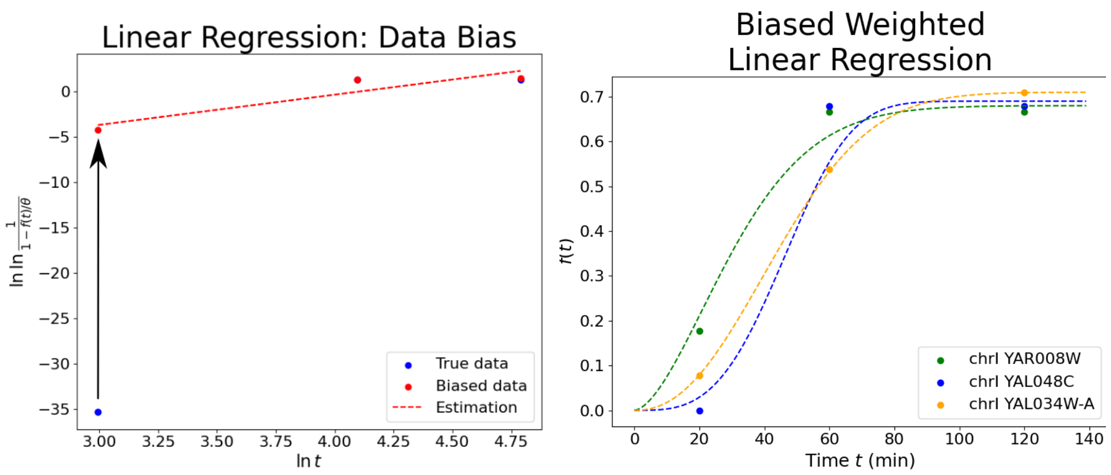

Fig 1 for Reviewers. (Left) By simply imposing strictly increasing repair, the original data (blue dots) are shifted (black arrow to red dots) such that the linear regression (red dashed line) in log space is simplified. (Right) This avoids a drastic switch-like behaviour for difficult data values. Blue is the *GEM1* gene example from the manuscript for which the data point at 20 minutes was rectified. Green shows the gene *SEN34* for which the time point at 120 minutes was corrected. Repair estimation for unproblematic regions remains unaffected (orange). Dots represent the data, whereas the dashed line gives the model approximation.

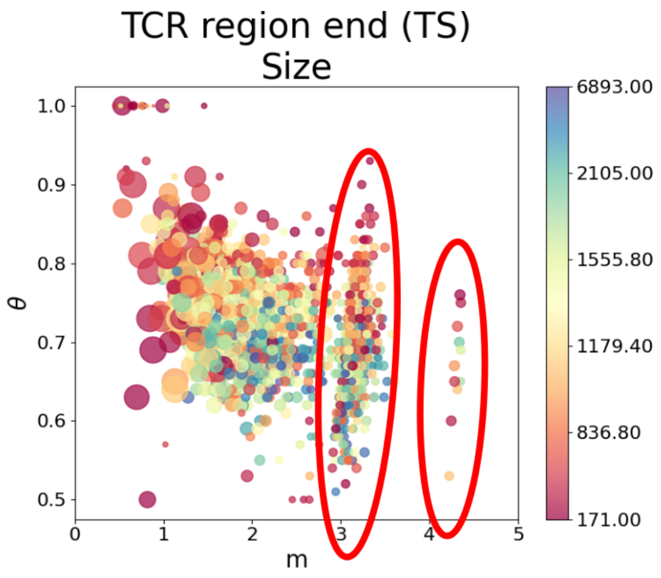

Fig 2 for Reviewers. The amended estimation method produces indeed acceptable parameter ranges even for difficult regions. However, due to the fact that they all include the same bias, they are clustered together in parameter space (red ellipses). They are therefore brought closely to the reliable parameter values (points outside the red ellipses). Points are coloured with respect to the transcription unit (TU) length. Red is small, whereas blue indicates large transcripts.

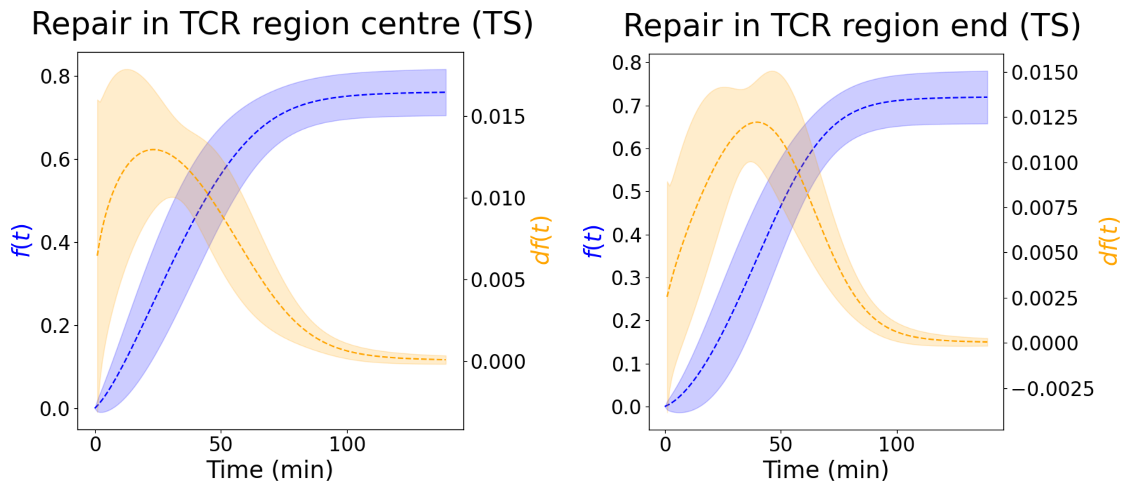

Fig 3 for Reviewers. Aggregated repair dynamics over a region type. The repair function is given in blue, and the derivative is depicted in orange. Dashed lines give the mean and shaded areas the standard deviation. (Left) Due to the fact that difficult regions are now closely located to reliable areas in parameter space, it becomes difficult to discern different repair kinetics for the TS centre. The clear effect of GGR in the centre of TCR regions vanishes through the changes in the algorithm. (Right) The different repair kinetics become distinguishable again for the TS end, where the standard deviation of the derivative shows two distinct peaks. However, they cannot be made visible by the mean. We gauge that an equal treatment of difficult and reliable regions is unjustified to produce clear results.

	***TCR* setup**							***Gene* setup**			
	**TS S**	**TS C**	**TS E**	**NTS S**	**NTS C**	**NTS E**	**NTCR**	**TS**	**NTS**	**IGR+**	**IGR-**
**NET**	s	s	s	i	i	i	-	s	s	-	-
**Size**	s	s	s	s	s	s	-	s	s	-	-
**ND**	**i**	s	s	s	s	s	i	s	i	s	s
**Abf1**	i	i	s	i	**s**	i	s	s	s	**s**	s
**H2A.Z**	s	s	s	s	s	s	s	s	s	**s**	**i**
**Meres**	i	i	i	i	i	i	**s**	s	**s**	**s**	**s**

Table 2 for Reviewers. Significant links between nuclear processes and repair. i denotes insignificant whereas s is significant. We defined a link to be significant if at least three out of five *k* find a non-random interrelationship. Non-random interrelationships that changed between the previous and amended algorithm are given in **bold**. Almost exclusively all influenced results occur in the gene configuration. - means that data was not used in the given configuration. NET denotes NET-seq data, ND is nucleosome density, and meres give the relative distance to centromeres or telomeres. Suffices S, C, and E denote start, centre and end of an area. NTCR are non-TCR areas. IGR are intergenic/non-transcribed regions.

We also want to report that we found a bug in the script for the model comparison. The prediction of the linear regression was accidentally compared to a constant rather than the actual data value, which created a large mean squared error. We corrected S16 Fig (C) accordingly. None of the conclusions were affected.

Finally, we want to thank Reviewer #2 for pointing out typos and some minor issues with the figure captions. In the following, we go through them step by step.

Reviewer #2:

1. I spotted a few possible typos when reading the revised manuscript:
a. Line 6: ‘evolutionary’ to ‘evolutionarily’?
b. Line 98: ‘lager’ to ‘larger’ ?
c. Line 152: ‘region’ to ‘regions’ ?

Answer:

Thanks for pointing them out, all of them were corrected.

Reviewer #2:

2. S3 Fig: Caption seems to be missing completely.

Answer:

We forgot indeed to add the S3 Fig caption in the appendix summary. This was added in the revised version.

Reviewer #2:

3. S4 Fig/Caption: The examples that are being used should be stated/described.

Answer:

We added some further description and stated the names of the used genes.

Reviewer #2:

4. S13 Fig Caption:
a. For panel B, for absolute clarity, that this is a single-gene example should be stated and the black dots need to be described as the experimental data points.
b. For panel C, again for absolute clarity, MSE should be defined and the meaning of the lines/bars and shaded regions in the plots should be described.

Answer:

We added an explanation for the used abbreviation MSE and a description of the used markers in the plots.

Reviewer #2:

5. S16 Fig/Caption:
a. The kind of correlation calculated should be stated/described somewhere
b. For clarity and ease of reading, the meaning of the numbers should be stated/described (also applies to S17 Fig Caption).
c. The units of the axes should be described (also applies to S17 Fig)

We added further information to the caption in S16 Fig and S17 Fig to address these issues.
